# Adolescent Development of Biological Rhythms in Female Rats: Estradiol Dependence and Effects of Combined Contraceptives

**DOI:** 10.3389/fphys.2021.752363

**Published:** 2021-11-05

**Authors:** Azure D. Grant, Linda Wilbrecht, Lance J. Kriegsfeld

**Affiliations:** ^1^The Helen Wills Neuroscience Institute, University of California, Berkeley, Berkeley, CA, United States; ^2^Department of Psychology, University of California, Berkeley, Berkeley, CA, United States; ^3^Department of Integrative Biology, University of California, Berkeley, Berkeley, CA, United States; ^4^Graduate Group in Endocrinology, University of California, Berkeley, Berkeley, CA, United States

**Keywords:** puberty, estrous, birth control, metabolism, signal processing, wavelet analysis

## Abstract

Adolescence is a period of continuous development, including the maturation of endogenous rhythms across systems and timescales. Although, these dynamic changes are well-recognized, their continuous structure and hormonal dependence have not been systematically characterized. Given the well-established link between core body temperature (CBT) and reproductive hormones in adults, we hypothesized that high-resolution CBT can be applied to passively monitor pubertal development and disruption with high fidelity. To examine this possibility, we used signal processing to investigate the trajectory of CBT rhythms at the within-day (ultradian), daily (circadian), and ovulatory timescales, their dependence on estradiol (E2), and the effects of hormonal contraceptives. Puberty onset was marked by a rise in fecal estradiol (fE2), followed by an elevation in CBT and circadian power. This time period marked the commencement of 4-day rhythmicity in fE2, CBT, and ultradian power marking the onset of the estrous cycle. The rise in circadian amplitude was accelerated by E2 treatment, indicating a role for this hormone in rhythmic development. Contraceptive administration in later adolescence reduced CBT and circadian power and resulted in disruption to 4-day cycles that persisted after discontinuation. Our data reveal with precise temporal resolution how biological rhythms change across adolescence and demonstrate a role for E2 in the emergence and preservation of multiscale rhythmicity. These findings also demonstrate how hormones delivered exogenously in a non-rhythmic pattern can disrupt rhythmic development. These data lay the groundwork for a future in which temperature metrics provide an inexpensive, convenient method for monitoring pubertal maturation and support the development of hormone therapies that better mimic and support human chronobiology.

## Introduction

Adolescence is a period of rhythmic reorganization during which physiology transitions from a non-reproductive juvenile state into reproductive early adulthood (Sisk and Foster, 2004; Hagenauer et al., 2011; Mohr and Sisk, 2013; Pereira et al., 2019). Historically, researchers have relied on self-report, Tanner staging, or infrequent salivary or blood hormone samples to track milestones of pubertal development. We reasoned that a time series characterization of continuous core body temperature (CBT) could reflect rhythmic features of hormonal development across puberty, as CBT exhibits clear rhythmic patterns that reflect underlying hormonal changes (Backstrom et al., 1982; Moenter et al., 1991; Smarr et al., 2016a; Grant et al., 2018, 2020b). If feasible, CBT could become a highly convenient and more accurate pubertal staging tool with unprecedented temporal resolution. Its application could also improve our understanding of the range of typical pubertal trajectories and factors that drive deviation from these trajectories.

Rhythmic development occurs at multiple timescales, including within-a-day (ultradian rhythms; URs; Bourguignon, 1988), daily (circadian rhythms; CRs; MacKinnon et al., 1978; Garcia et al., 2001), and multi-day ovulatory cycles in females (ovulatory rhythms; ORs; Vidal, 2017). These rhythms occur across physiological systems, serving to increase the efficiency of signal transduction (Lloyd and Stupfel, 1991; Brodsky and Lloyd, 2008; Walker et al., 2010), temporally segregate incompatible processes (Panda, 2016), synchronize internal systems to the environment (Dibner et al., 2010), and maximize reproductive success (Carlson and Shaw, 2019). Although structure at one timescale can be modulated by changes at another (Backstrom et al., 1982; Shechter and Boivin, 2010), distinct mechanisms underlie each rhythmic frequency. This suggests individual rhythmic frequencies may each follow distinct developmental trajectories.

Coordinated URs are observed in hypothalamic-pituitary-peripheral axes and beyond in adult mammals, with broad manifestation in systems including cardiovascular outputs, thermoregulation, and even cognition (reviewed in: Brandenberger et al., 1987; Shannahoff-Khalsa et al., 1996; Brodsky, 2014; Grant et al., 2018; Goh et al., 2019). Many URs, including those in reproductive and growth hormones, are present early in life (Ojeda et al., 1986) and increase in amplitude from pre to mid puberty (Dunger et al., 1991a; Albertsson-Wikland et al., 1997; Apter, 1997). Some URs increase markedly in frequency and amplitude around puberty onset [e.g., gonadotropin releasing hormone (GnRH), luteinizing hormone (LH), and estradiol (E2); Jenner et al., 1972; Bourguignon, 1988; Dunger et al., 1991a,b; Apter et al., 1994; Ojeda and Skinner, 2006; Shaw et al., 2012]. In adults, URs are modulated by time of day (Refinetti, 1994) and phase of the ovulatory cycle (Backstrom et al., 1982), suggesting that pubertal modifications at the CR and OR timescales likely impact UR structure. Although, URs are thought to be centrally controlled, potentially *via* interaction of dopaminergic and hypothalamic circuits (Ootsuka et al., 2009; Merkley et al., 2012, 2015; Blum et al., 2014; Prendergast and Zucker, 2016; Goh et al., 2019; Lehman et al., 2019), relatively little is understood about mechanisms and progression of URs across adolescence.

Whereas the mechanisms and phenomenology of ultradian development require much further study, those underlying circadian rhythms and pubertal changes to CRs are well-documented. Circadian rhythms are nearly ubiquitous, generated intracellularly *via* interlocked transcription-translation feedback loops, and are governed by a central pacemaker within the suprachiasmatic nucleus (SCN) of the hypothalamus (Hagenauer et al., 2011; Takahashi, 2017; Hastings et al., 2018). Although changes in SCN output and connectivity during adolescence are not well-characterized, integration of new neurons into the central clock (Mohr et al., 2017) and reproductive neurocircuitry (Mohr et al., 2019) alongside adolescent increases in SCN input to the GnRH system (Kriegsfeld et al., 2002) may contribute to downstream CR changes. For example, circadian amplitude appears to increase across puberty in many systems (e.g., cortisol; Duan et al., 2018, activity; Hagenauer et al., 2011, and potentially temperature; Pronina et al., 2015), while emerging for the first time in others (e.g., LH; Ojeda and Skinner, 2006 and FSH; Albertsson-Wikland et al., 1997). Finally, circadian activity (Hagenauer et al., 2011) and sleep-wake (Hummer and Lee, 2016) rhythms are phase delayed during puberty (Hagenauer and Lee, 2012) and are more vulnerable to disruption by mistimed light and food cues than in adults (Crowley et al., 2015).

In contrast to ultradian and circadian rhythms, which are apparent to variable degrees in juveniles, the female ovulatory, or estrous, cycle emerges for the first time in adolescence (Vidal, 2017). Briefly, in spontaneously-ovulating rodents, the 4–5day cycle begins with rising E2 levels that maintain LH at low concentrations through negative feedback. When E2 is sufficiently high, and a pool of ovarian follicles has matured, E2 positive feedback integrates with circadian signaling and progesterone of neural origin (Kim et al., 2018; Angelopoulou et al., 2019; Mohr et al., 2019) to induce a preovulatory LH surge that initiates ovulation (Gibson et al., 2008; Williams et al., 2011; Ginther et al., 2013; Piet et al., 2015; Russo et al., 2015; Angelopoulou et al., 2019). Subsequent formation of the corpus luteum leads to a brief rise in circulating progesterone prior to beginning the next cycle. As with URs and CRs, ORs manifest as changes in thermoregulation with E2 decreasing temperatures prior to ovulation, and E2 with progesterone increasing temperature following ovulation (Reviewed in: Webster and Smarr, 2020). Although it is clear that ORs emerge at puberty, the continuous patterns of commencement and stabilization are poorly understood. Pre-pubertal ovarian follicles typically undergo development and atresia without substantial release of sex steroids (Apter, 1997). Soon after the emergence of the first cycle, at menarche in girls (Carlson and Shaw, 2019), cycles have a higher likelihood of anovulation or low post-ovulatory progesterone compared to adults (Peña et al., 2018). Although, high temporal resolution patterns are unknown, large increases in plasma E2 and FSH occur from pre to mid puberty (Delemarre-Van De Waal et al., 1991; Dunger et al., 1991a; Ojeda and Skinner, 2006). Given these changes in hormones across puberty, one aim of the present investigation was to employ continuous, longitudinal, and high-resolution CBT monitoring alongside daily E2 measures to characterize the emergence of the ovulatory cycle in rats.

By characterizing rhythmic outputs that reflect underlying physiological change across adolescence, a greater understanding of typical progression can be garnered and the impact of exogenous hormone manipulation on temporal trajectories can be observed. Temporal disruption at all three timescales is associated with negative health outcomes in adults (Gibson et al., 2010; Casper and Gladanac, 2014; Gotlieb et al., 2018; Kalafatakis et al., 2018; Lightman et al., 2020; Wang et al., 2020), and adolescence may be a sensitive period, where disruptions have rapid (Gupta and Khare, 2020) and potentially long-term health impacts (Carskadon et al., 2002; Crowley et al., 2015; Logan et al., 2018; Pereira et al., 2019). Female hormonal contraception is a common example of such disruption in adolescence. A growing proportion of teenage girls (estimated between 22 and 54% across the first two decades of the 21st century; Birth Control Pill Use-Child Trends, 2018) receive hormonal contraceptives for a variety of purposes, including pregnancy prevention (Apter, 2018), treatment of menstrual symptoms (Adeyemi-Fowode et al., 2017), and acne (Mwanthi and Zaenglein, 2018). As hormonal contraceptives are delivered at static or once daily bolus concentrations that differ from the endogenous, multiscale rhythmic pattern of release (Naqvi et al., 1984; Zhou et al., 1998; Strom et al., 2010), these drugs can be considered a form of temporal endocrine disruption (Landersoe et al., 2020; Lucaccioni et al., 2020). Although currently considered safe, discontinuation rate is high (Coukell and Balfour, 1998) and impact on the temporal progression of development is unclear.

The present study employed continuous CBT to characterize rhythmic change across adolescent development and examine the role of pubertal onset of E2 production in guiding the typical developmental trajectory. Additionally, because late pubertal contraceptive use might act to disrupt the typical progression of rhythmic developmental changes, we examined the impact of a common contraceptive regimen [i.e., ethinyl E2 (EE2) and levonorgestrel] on endogenous estradiol concentrations and CBT rhythms. As the pulse amplitude of multiple hormones increases across adolescence, we hypothesized that the amplitude of CBT URs would be similarly impacted. We also hypothesized that CR amplitude and overall body temperature would increase during adolescence, and that these increases would be influenced by E2. As changes to rhythmicity have primarily been reported from early to mid-adolescence, we hypothesized that rhythmic restructuring would be most pronounced during this period. Finally, we hypothesized that rhythmic patterns of body temperature change identified during adolescent development would be disrupted during and potentially after, the cessation of contraceptive administration.

## Materials and Methods

### Animals

Female and Male Wistar rats were purchased at 250 and 300g, respectively, from Charles River and bred in the lab. Pups were weaned at p21, with a maximum of one pup pair (one experimental and one partner pup) contributed to each experimental group per litter. Weanlings were housed in standard translucent propylene (96×54×40cm) rodent cages, and provided *ad libitum* access to food and water, wood chips for floor cover, bedding material, and chew toys for the duration of the study. To minimize social isolation stress, which is known to affect pubertal development (Bakshi and Geyer, 1999; Boggiano et al., 2008), animals were housed with a same sex, non-experimental sibling. Animals were maintained on a 12:12 light dark (LD) cycle; light intensity during the photo- and scotophases were ~500lux white light and <1lux red light, respectively, with lights on at 1AM and off at 1PM (Brainard, 1988; Zhang et al., 2017). Animals were gently handled before weighing daily to minimize stress. To prevent mixing of feces used for hormone analysis, cage mates were separated by a flexible stainless steel lattice that permitted aural, scent, and touch interaction between siblings. A total of 64 animals were included in the study: 32 as experimental animals [Intact, Intact+Contraceptives, Ovariectomized (OVX) and OVX+E2; *n*=8/group], and 32 as social, littermate partners. All procedures were approved by the Institutional Animal Care and Use Committee of the University of California, Berkeley.

### Core Body Temperature Data Collection

Data were gathered with G2 E-Mitter implants that chronically record CBT (Starr Life Sciences Co., Oakmont, PA, United States). At weaning, G2 E-Mitters were implanted in the intraperitoneal cavity under isoflurane anesthesia with analgesia achieved by subcutaneous injections of 0.03mg/kg buprenorphine (Hospira, Lake Forest, IL, United States) in saline (administered every 12h for 2days after surgery). E-Mitters were sutured to the ventral muscle wall to maintain consistent core temperature measurements. Recordings began immediately, but data collected for the first 4days post-surgery were not included in analyses. Recordings were continuous and stored in 1-min bins.

### Ovariectomy and Silastic Capsule Replacement

Ovariectomies were performed at weaning (p21) at the same time as the implantation of the E-Mitter, as previously described (Russo et al., 2015; Smarr et al., 2017). The E-Mitter surgery served as a control operation in non-OVX animals. Incisions were closed using dissolvable sutures and wound clips. At p29, OVX animals were anesthetized and implanted with silastic capsules (0.78mm I.D., 1.25 O.D.; Dow Corning, Midland, M). Capsules were implanted subcutaneously and intrascapular. Capsules were 20mm in length with 5mm silicone sealant (Sigma Aldrich, St. Louis, MO, United States) at each end and contained either 112μg (180μg/ml) 17β estradiol (Fisher Scientific, Hampton, NH) in sesame oil, or sesame oil alone. E2 treatment results in plasma E2 concentrations averaging ~5μg/day, beginning in animals of 80–100g (Andrews et al., 1981; Ström et al., 2012). Capsules were primed for 24h prior to implantation *via* submersion in 0.9% saline at 25°C in order to avoid delivering a large initial bolus of E2. As data on temperature following the implant of the capsule were not analyzed until the wound healing from the capsule implant had occurred, transient temperature changes arising from any initial implantation elevation in estradiol would not have impacted the analyses presented here. Although these doses have been tested previously, large variability in serum E2 levels following silastic implant is typically reported (Andrews et al., 1981; Clark and Tarttelin, 1982; Day et al., 1986; Ström et al., 2012). Incisions were closed using dissolvable suture and a wound clip, and buprenorphine was delivered as above for post-operative analgesia.

### Contraceptive Administration

Ethinyl Estradiol (30μg/day; Fisher Scientific) and Levonorgestrel (30μg/day; Fisher Scientific), a progestin, were dissolved in 0.01ml of sesame oil and delivered subcutaneously at the nape of the neck daily for 8days, the approximate duration of two estrous cycles, during mid to late adolescence (p50–p58), with control animals receiving vehicle. Although, a wide range of rodent doses of EE2 and Levonorgestrel have been reported (Santoru et al., 2014; Prakapenka et al., 2018; Olaniyi and Olatunji, 2019), the doses chosen here aimed to match those used consistently for suppressing ovulation and mimicking effects observed in humans, such as increased blood pressure (Geraghty et al., 1990), and for comparability to existing rodent literature on subcutaneous delivery of Levonorgestrel and EE2 (Follesa et al., 2002; Simone et al., 2015). Many doses for orally delivered EE2 and Levonorgestrel in rats also fall in this range (Nowaczyk-Dura and Czekaj, 1998; Guerra et al., 2002; Olatunji et al., 2017; Olaniyi and Olatunji, 2019). These drugs have been available as human contraceptives for decades under several brand names in widely varying doses [JADELLE, n.d.; Levonorgestrel And Ethinyl Estradiol (Oral Route) Description and Brand Names-Mayo Clinic, n.d.]. We elected not to standardize dose by body mass to mimic the human condition, where dose of contraceptive is not standardized by weight in teens.

### Fecal Sample Collection

Fecal E2 (fE2) concentrations were assessed across puberty from feces generated over 24h periods. Fecal samples provide hormone concentrations more representative of average daily hormone concentrations than single timepoint blood samples (Harper and Austad, 2000; Millspaugh and Washburn, 2003; Touma et al., 2004; Woodruff et al., 2010; Auer et al., 2020) and eliminates associated stress and infeasibility of high-frequency, longitudinal blood collection. Samples were collected in small airtight bags at the end of dark phase under dim red light (<5lux) from a minimum of p25–p37 (pre puberty and first cycle), p45–p51 (mid-puberty), and p55–p65 (late puberty to early adulthood) in all groups, and additionally to p75 in Intact+C and Intact groups (adulthood). Samples soiled with urine were discarded, and all other droppings generated over each 24-h segment were combined. Within 1h of collection, samples were stored at −20°C until processed. Sample collection was rapid (~1min per animal). Before assessment of hormone concentration, samples were processed according to manufacturers’ instructions. Briefly, samples were placed in a tin weigh boat and heated at 65°C for 90min, until completely dry. Dry samples were ground to a fine powder in a coffee grinder, which was wiped down with ethanol and dried between samples to avoid cross contamination. Powder was weighed into 0.2mg aliquots. For hormone extraction, 1.8ml of 100% ethanol was added to each test tube, and tubes were shaken vigorously for 30min. Tubes were then centrifuged at 5,000 RPM for 15min at 4°C. Supernatant was moved to a new tube and evaporated under 65°C until dry (~90min). Sample residue was reconstituted in 100μl of 100% ethanol. Around 25μl of this solution was diluted for use in the assay and remaining sample was diluted and stored.

### ELISA Assays

A commercially available fE2 ELISA kit was used to quantify E2 in fecal samples (Arbor Assays, Ann Arbor, MI, United States). These assays have been previously published in species ranging from rats and mice (Mathew et al., 2017; Lv et al., 2020), to wolves (Franklin et al., 2020), to humans (Righetti et al., 2020). ELISAs were conducted according to manufacturer’s instructions. To ensure each sample contained ≤5% alcohol, 25μl of concentrate were vortexed in 475μl assay buffer. All samples were run in duplicate, and an inter-assay control was run with each plate. Sensitivity for the assay was 39.6pg/ml and the limit of detection was 26.5pg/ml. fE2 intra-assay coefficient of variation (COV) was 5.94% and inter-assay COV was 5.71%.

### Data Availability and Analysis

All code and data used in this paper are available at A.G.’s and L.J.K.’s Github Repository (azuredominique, 2021; Kriegsfeld-Lab, 2021).^1^ Code was written in MATLAB 2020b with Wavelet Transform (WT) code modified from the Jlab toolbox and from Leise (2013, 2015). Briefly, data were imported to MATLAB at 1-min resolution. Any data points outside ±4 SDs were set to the median value of the prior hour, and any points showing near instantaneous change, as defined by local abs (derivative) >10^5^ as an arbitrary cutoff, were also set to the median value of the previous hour. Small data gaps resulting from intermittent data collection (<10min) were linearly interpolated. Continuous data from p26 to p74 were divided into three equal-length phases: early to mid-puberty (p26–p41), mid to late puberty (p42–p58), and late puberty to early adulthood (p59–p74).

### Wavelet Analyses and Statistics of CBT Data

Wavelet Transformation was used to generate a power estimate, representing amplitude and stability of oscillation at a given periodicity, within a signal at each moment in time. Whereas Fourier transforms allow transformation of a signal into frequency space without temporal position (i.e., using sine wave components with infinite length), wavelets are constructed with amplitude diminishing to 0 in both directions from center. This property permits frequency strength calculation at a given position. In the present analyses, we use a Morse wavelet with a low number of oscillations (defined by *β*=5 and *γ*=3, the frequencies of the two waves superimposed to create the wavelet; Lilly and Olhede, 2012), similar to wavelets used in many circadian and ultradian applications (Lilly and Olhede, 2012; Leise, 2013, 2015; Smarr et al., 2016b, 2017; Grant et al., 2020a). Additional values of *β* (3–8) and *γ* (2–5) did not alter the findings (data not shown). As WTs exhibit artifacts at the edges of the data being transformed, only the WT from p26 to p74 were analyzed further. Periods of 1–39h were assessed. For quantification of spectral differences, WT spectra were isolated in bands; circadian periodicity power was defined as the max power per minute within the 23–25h band, consistent with previous work establishing that this band captures the circadian range of all rats of this strain housed in a light:dark cycle (Hagenauer et al., 2011; Hagenauer and Lee, 2012); ultradian periodicity power was defined as the max power per minute in the 1–3h band. The latter band was chosen because this band corresponded with the daily ultradian peak power observed in URs across physiological systems in rats (Kottler et al., 1989; de Kloet and Sarabdjitsingh, 2008; Sanchez-Alavez et al., 2010; Grant et al., 2018).

For statistical comparisons of any two groups, Mann Whitney U (MW) rank sum tests were used to avoid assumptions of normality for any distribution. Non-parametric Kruskal-Wallis (KW) tests were used instead of ANOVAs for the same reason; for all tests, *χ*^2^ and *p* values are listed. In cases of repeated measures within an individual over adolescence, Friedman’s tests were used. Data from each estrous cycle were treated as independent. We chose to treat estrous cycles as independent for three reasons: (1) the estrous cycle is the longest periodicity rhythm within the study, (2) the adolescent estrous cycle develops rapidly from one iteration to the next in rats, and (3) because the interventions of ovariectomy and birth control administration exert their effects by removal or modification of estrous cycles. Dunn’s test was used for multiple comparisons. Mann Kendall (MK) tests were used to assess trends over time in wavelet power and linear CBT over three equally sized temporal windows described above. For short term (<3days of data) statistical comparisons, 1 data point per hour was used; for longer term (>3days of data) statistical comparisons, 1 data point per day was used. Continuous wavelet power data were smoothed with a 24h window using the MATLAB function “movmean.” Violin plots, which are similar to box plots with probability density of finding different values represented by width (Violin Plots 101: Visualizing Distribution and Probability Density, 2016), were calculated using the MATLAB function “violin.” Median daily circadian power was regressed against each day’s fE2 for each group using a mixed effects linear regression [MATLAB function “fitlme,” formula: circadian daily medians~1+E2 values+(1+E2|Individual ID)].

### Data Alignment and Analysis of fE2 Concentrations and Estrous Cyclicity

As all animals do not begin puberty on the same day of life, group alignment of estrous cycles was conducted by grouping cycles since commencement of puberty, for each individual, as assessed by fE2. Briefly, for each animal, fE2 was assessed in 4-day blocks. During each block, fE2 rose over 3 subsequent days with a decrease on the fourth. E2 cycles were aligned across animals using the peak value on day 3. For example, if animal one began puberty on p30 and exhibited a 4-day window peak of fE2 on p33, then that animal’s “first cycle” would be displayed and averaged into a group representation of first cycle as p31, p32, p33, and p34. This strategy enabled group assessment of a pre-pubertal 4-day window, as well as an early, mid, and late pubertal cycle, and an early adulthood cycle for Intact and Intact+C animals.

The day of fE2 rise before cycling began was defined as the first day fE2 level rose >2 SDs above its starting value at p25. The initial rise in fE2 was used as an alignment point for CBT, ultradian power, and Z-Score (CBT) – Z-Score (UR power) group averages. Group differences in fE2 area under the curve by cycle were assessed using the MATLAB function “trapz” and KW tests with Dunn’s *post hoc* correction. As estrous cycles are not all aligned in time or by age, samples were aligned with the highest value in a collection period (e.g., mid puberty), where a “fall” was observed 3days later. Fast Fourier Transforms (FFT) were used to assess the presence or absence of 4–5day power in CBT in each individual from the period of fE2 rise until p50 (when Intact+C animals started receiving daily contraceptive injections), and from p50 to p74. In order to further assess commencement and stability of estrous cycling after first rise in fE2, as well as any potential perturbation during and after contraceptive administration, metrics were divided into 4day blocks, with each day labeled 1, 2, 3, and 4: repeating for subsequent cycle lengths. Groups for statistical comparison were constructed from all data corresponding to 1’s, 2’s, 3’s, and 4’s. Friedman’s tests with Dunn’s correction for multiple comparisons were used to determine if values associated with each day of cycle (e.g., all day 1’s) varied significantly from other days of the cycle by group.

## Results

### Impact of Hormonal Status on Estradiol Concentrations and Weight Gain Across Adolescence

**Figure 1:**
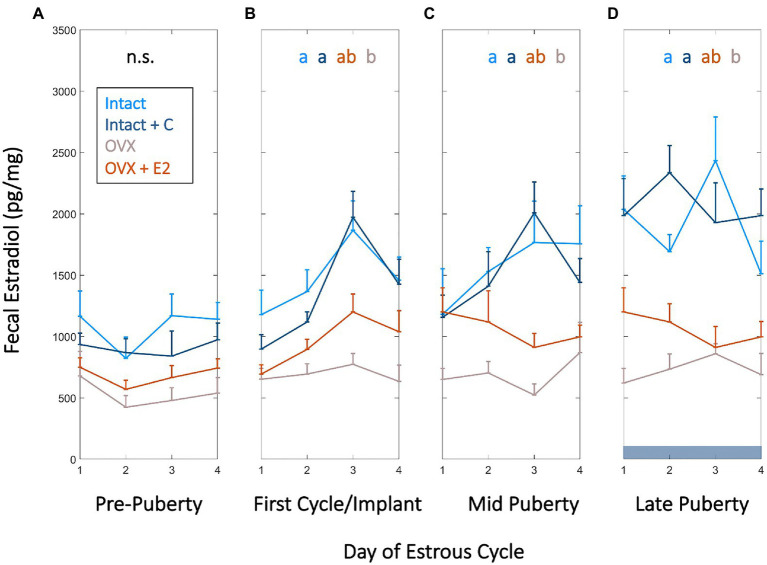
High frequency fecal estradiol (fE2) enables monitoring of estrous cycle emergence, sex steroid manipulation, and ovarian status during adolescence. Group mean (+SEM) fE2 concentrations of Intact (light blue), Intact+short-term pubertal contraceptives (Intact+C; dark blue), Ovariectomized (OVX; gray), and OVX+E2 (orange) groups did not significantly differ prior to puberty (p24–p30; **A**). Fecal estradiol in Intact and Intact+C groups increased over that of OVX animals beginning at the first cycle following vaginal opening or relative to the time of silastic implant in OVX+E2 animals (p30–p37; **B**) and remained significantly elevated thereafter at mid puberty (p43–p49; **C**) and during late puberty (p55–p61; **D**). Dark horizontal bar in **D** indicates Intact+C data were gathered during contraceptive administration. Color of letters at the top of **B**,**C**, and **D** indicate experimental group; letters indicate statistical differences, with groups not sharing the same letter being significantly different (*p*<0.03).

Frequent fE2 measurements were collected to assess if hormonal status affected the level or temporal patterning of fE2 across puberty. FE2 concentrations did not differ between groups from p25 to p31, a baseline period prior to puberty onset (*χ*^2^=4.48, *p*=0.214; Figure 1A). Vaginal opening occurred between p31 and p33 in Intact rats, and fE2 rose 2 SDs above its p25 starting value between p31 and p36.

During this window, Intact and Intact+C animals’ fE2 concentrations exceeded that of OVX animals (*χ*^2^=15.9, *p*=0.001; *p*=0.0134 and *p*=0.003, respectively; Figure 1B). This difference was maintained at mid puberty (cycles aligned from p40 to p47; *χ*^2^=13.7, *p*=0.003; *p*=0.003 and 0.032, respectively) and early adulthood (cycles aligned from p55 to p61; *χ*^2^=17.1, *p*=0.001; *p*=0.001 and 0.009, respectively; Figures 1C,D). OVX+E2 animals were not different from other groups at any timepoint, with intermediate values between Intact and OVX groups (*p*>0.05 in all cases). Unlike Intact animals, Intact+C animals did not exhibit days of elevated fE2 every 4th day (See Supplementary Figure 1). However, fE2 concentrations did not differ between Intact and Intact+C groups approximately 4–5cycles after contraceptive administration ceased, between p69 and p75 (*χ*^2^=1.62, *p*=0.203). See section “Materials and Methods” for details of within-cycle alignment.

Additionally, daily weights were measured to recapitulate known effects of E2 on pubertal growth trajectory, and to assess if contraceptive administration modulated weight gain. Intact animals gained weight consistently across puberty. Pre-pubertal OVX was associated with increased body weight at mid puberty, with OVX animals weighing more than animals from all other groups, and OVX+E2 animals weighing more than Intact or Intact+C groups (*χ*^2^=57.9, *p*=1.65*10^−12^; *p*<0.05 for all individual comparisons). By early adulthood, both OVX and OVX+E2 groups weighed significantly more than Intact or Intact+C groups and did not differ from one another (*χ*^2^=76.1, *p*=2.08*10^−16^; *p*=0.99 for OVX vs. OVX+E2; *p*<0.05 for all other comparisons). Eight days of contraceptive administration did not significantly impact weight relative to Intact animals (*χ*^2^=0.28, *p*=0.594; See Supplementary Figure 2).

### Circadian, but Not Ultradian, Power of Body Temperature Increased Across Pre to Mid Adolescence

**Figure 2:**
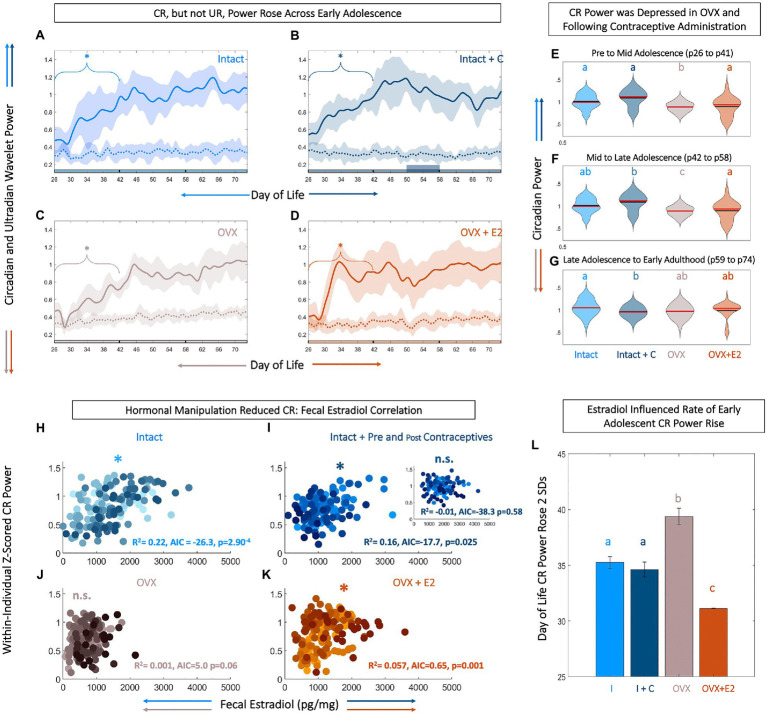
Level and rate of early adolescent rise in core body temperature (CBT) circadian power are tied to fecal estradiol (fE2). Circadian, but not ultradian power rises across early adolescence **(A-D)**. Linear plots of group mean (±SD) of CBT circadian (solid) and ultradian (dashed) power in Intact (light blue, **A**), Intact+C (dark blue, **B**), OVX (gray, **C**), and OVX+E2 (orange, **D**) animals. * indicates significant trend over time for the bracketed time region (*p*<0.05). Phase of adolescent time periods (pre to mid, mid to late, and late to adult) are indicated by black dividers in the colored x-axis at p42 and p58. Although, CBT circadian power rises over early adolescence in all groups, estradiol increases the rate of this rise **(L)**. Violin plots **(E–G)** of circadian power in each group analyzed by segment of life: pre to mid adolescence from p26 to p41 **(E)**, mid to late adolescence from p42 to p57 **(F)**, and late adolescence through early adulthood from p58 to p73 **(G)** illustrate that circadian rhythm (CR) power is highest in Intact animals, with a reduction following hormonal contraceptive administration and OVX, and a partial rescue in OVX+E2 animals. Black lines indicate mean and red lines indicate median of each plot. Scatters of fE2 level by CR power indicate that hormonal manipulation reduces or eliminates the correlation between CR power and fE2 concentrations **(H-K)**. Note that each individual within a group is plotted in a unique color. CR power and fE2 are significantly correlated in Intact (light blue, **H**), Intact+C animals prior to contraceptive administration (dark blue, **I**), with inset depicting abolished correlation during and after contraceptive administration. CR power and fE2 are weakly correlated in OVX+E2 (orange, **K**) but not OVX animals **(J)**. * indicates significant positive correlation between fE2 and CR power. AIC indicates relative performance of the mixed effects model. Color of letters at the top in **E–G** and **L** indicate experimental group; letters indicate statistical differences, with groups not sharing the same letter being significantly different (*p*<0.03).

As reproductive circadian and ultradian rhythms change markedly across adolescence and may be coupled to CBT (Wu et al., 1996; Shaw et al., 2012; Duan et al., 2018), we investigated the impact of estradiol status on the timing and tempo of CBT rhythmicity. All animals exhibited a significant positive trend in CR power from pre-to-mid adolescence (p26–p41; *p*=0.002, 0.007, 0.029, and 0.04 for Intact, Intact+C, OVX, and OVX+E2 animals, respectively). CR power stabilized thereafter (*p*>0.05 in all cases; Figures 2A,B). To examine the relative rate of this increase across groups, we set a criterion of 2 SDs above the mean. CR power rose 2 SDs above the mean significantly faster in OVX+E2 animals compared to Intact or OVX animals (*χ*^2^=19.0, *p*=3*10^−4^; *p*=0.025 and 0.001, respectively; Figure 2L).

Ultradian power did not show a significant upward or downward trend across the study period in any group (*p*>0.05 for all groups at all time windows; Figures 2A–D). Although directionality of CR power change across adolescence was similar (i.e., an early increase followed by a plateau) in all individuals, magnitude of circadian power differed among groups. Specifically, circadian power for OVX animals was depressed compared to all other groups from pre to mid adolescence (*χ*^2^=125, *p*=4.02*10^−27^, *p*<0.02 for OVX vs. all other groups; Figure 2E). From mid to late adolescence (p42–p58), CR OVX power remained depressed and OVX+E2 trended toward lower power (*χ*^2^=112, *p*=2.82*10^−24^; *p*<0.01 Intact and Intact+C vs. OVX; Figure 2F). In early adulthood (p59–p74), following contraceptive administration, the CR power for Intact+C was depressed compared to Intact rats (*χ*^2^=37.9, *p*=2.93*10^−08^, *p*=0.04; Figure 2G).

### Body Temperature Circadian Power Was Most Correlated to Fecal Estradiol Level in Unmanipulated Animals

Temperature level, UR power, and E2 exhibited coupled patterning, and appeared to change markedly during adolescence. However, it was unclear if CBT circadian rhythmicity was coupled to estradiol, if such a relationship existed during adolescence, and if the relationship could be modified by hormonal state. FE2 and normalized circadian power were strongly correlated in Intact (*R*^2^=0.226, *p*=2.90*10^−4^) and Intact+C rats prior to p50 when contraceptive administration began (*R*^2^=0.166, *p*=0.025), and weakly correlated in OVX+E2 animals (*R*^2^=0.062, *p*=0.001; Figures 2H–K). This positive correlation was abolished during and after hormonal contraceptive administration in the Intact+C group (*R*^2^=1.06*10^−5^, *p*=0.58; Figure 2I, inset). Circadian power and fE2 was not significantly correlated in OVX animals (*R*^2^=0.013, *p*=0.06; Figure 2J).

### Ovulatory Rhythms in CBT and Perturbations During and After Contraceptive Administration

**Figure 3:**
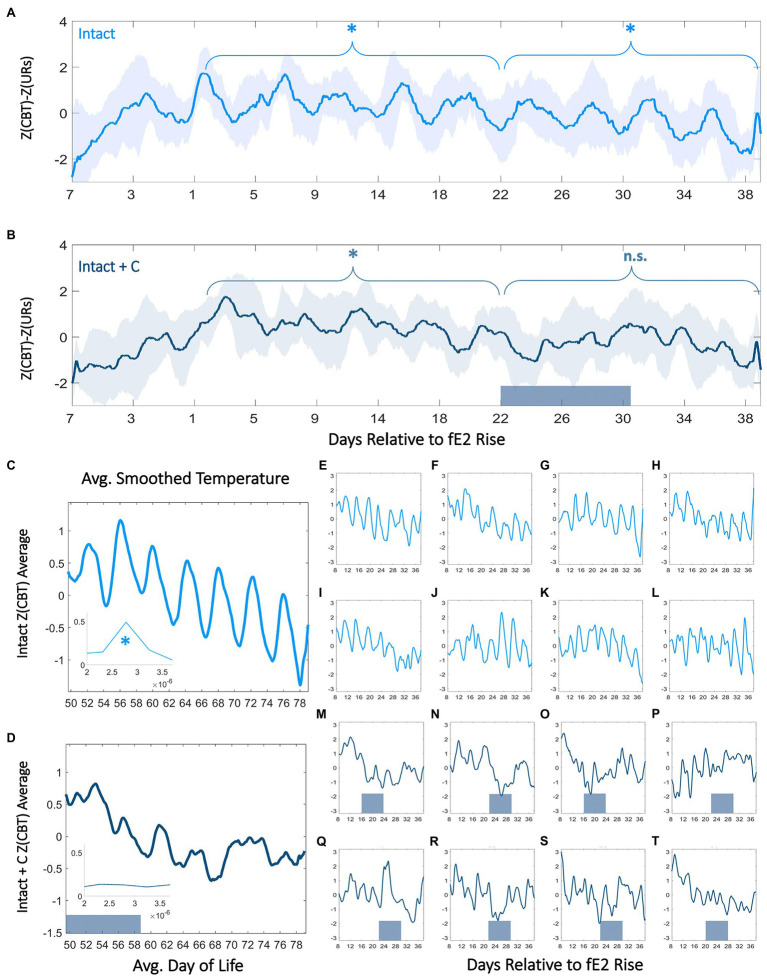
Contraceptive administration in adolescence persistently perturbs 4-day temperature rhythms. Normalized CBT mean (±SD) minus ultradian rhythm (UR) power relative to within-individual first day of fE2 rise in Intact **(A)** and Intact+C **(B)** rats (see: Materials and Methods and Supplementary Figure 3). Dark bars along the x-axis for Intact+C animals indicate average time of contraceptive administration relative to fE2 rise. * indicates regions of time over which every 4th day’s CBT values are significantly elevated compared to other days of cycle (*p*<0.003). Twenty-four hour smoothed average plots of normalized linear CBT in Intact **(C)** and Intact+C **(D)** individuals from the time of contraceptive administration illustrate a reduction in regularity of 4-day oscillations. Insets show FFT centered at 4–5days. * indicates significantly higher AUCs in the 4–5day range for Intact (panel **C**) compared to Intact+C rats (panel **D**). Individual animals **(E–T)** comprising Intact (light blue) and Intact+C (dark blue) groups prior to and following hormonal contraceptive administration. Dark bars along horizontal axes indicate time of contraceptive administration; administration days differ based on an individual’s day of fE2 rise.

**Figure 4:**
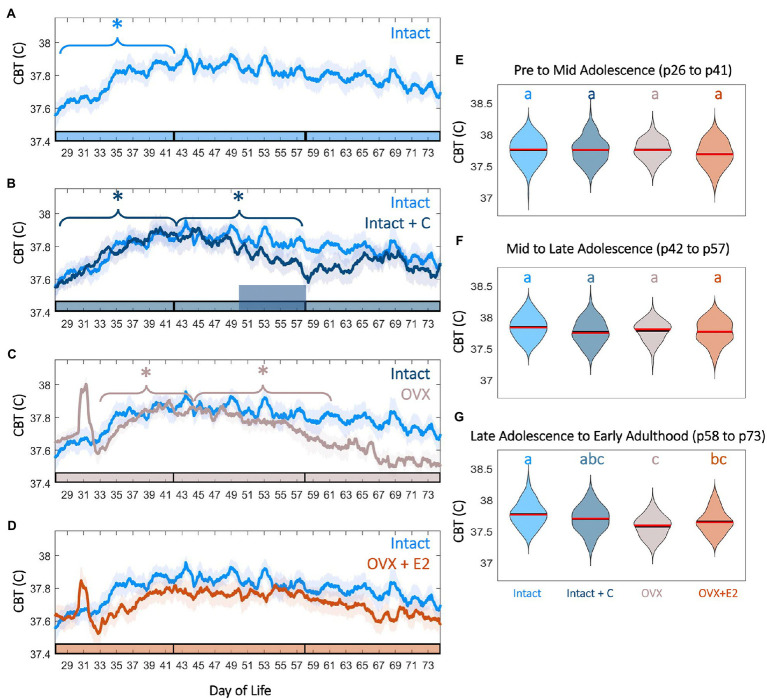
Female adolescence is associated with sex steroid-dependent CBT levels and trends. CBT linear group means (±SD) in Intact (**A**, light blue) compared to Intact+C (**B**, dark blue), OVX (**C**, gray), and OVX+E2 (**D**, orange) animals. * indicates significant trend during the bracketed time period for the group matching the color of the bracket (*p*<0.03). Phase of adolescence time periods (pre to mid, mid to late, and late to adult) are indicated by breaks in the colored x-axis at p42 and p58. Time of hormonal contraceptive administration in panel **B** is indicated by the tall horizontal bar. Violin plots of temperature for all groups at early to mid adolescence **(E)**, mid to late adolescence **(F)**, and early adulthood **(G)** indicate that hormonal contraceptive administration leads to reductions in CBT relative to controls after administration. Ovariectomy, even with E2 replacement, is also associated with significantly reduced temperatures by early adulthood **(G)**. Color of letters at the top in **E–G** indicate experimental group. Letters indicate statistical differences, with groups of different letters being significantly different (*p*<0.001).

We next investigated the relationship between ORs and cycles in body temperature. Our goal was to determine if interactions at this timescale (a) could be observed in continuous body temperature in adolescents, (b) if previously described hormonal UR modulations by phase of cycle (Gabriel et al., 1992; Hoeger et al., 1999) translates to ovulatory cycles in CBT URs, and (c) if exogenous hormone administration disrupts these continuous dynamics. In intact rats, we observed a significant 4-day modulation of combined CBT and UR power corresponding to the estrous cycle (Intact *χ*^2^=59.1, *p*=9.26*10^−13^, Intact+C group prior to BC administration *χ*^2^=13.7, *p*=0.003; Figures 3A,B, 4A,B). This 4-day pattern commenced in Intact and Intact+C rats (prior to treatment) with a significant increase in mean daily CBT following the first rise in fE2 above 2 SDs (*p*=0.03 in each case). Intact and Intact+C (prior to treatment) animals also exhibited a 4-day pattern of UR power modulation. This modulation manifested as a significant trough of UR power within 4days of the first rise of fE2, as previously reported in adult rodents (Sanchez-Alavez et al., 2011; Prendergast et al., 2012; Smarr et al., 2016b, 2017; *p*=0.04, *p*=0.03, respectively). The combination of UR power and linear temperature yielded a more easily separable metric, which rose significantly the day after first rise of fE2 in Intact and Intact+C (*p*=0.01, *p*=0.02, respectively; Figures 3A,B; For individual metric comparisons see Supplementary Figure 3).

We noted an absence of significant 4-day differences in combined CBT and UR power in the Intact+C group during hormonal contraceptive administration, even following 4cycle-lengths of recovery (Intact+C group *χ*^2^=7.2, *p*=0.07; Intact group over same time period *χ*^2^=58.9, *p*=1.00*10^−12^). A FFT of data in Intact and Intact+C animals prior to contraceptive administration revealed comparable AUCs for 4–5day oscillations (no group difference; *χ*^2^=0.54, *p*=0.46; Figures 3C,D, Inset). However, after hormonal contraceptive administration, AUC for Intact animals was significantly greater for 4–5day oscillations than in Intact+C animals (*χ*^2^=3.98, *p*=0.046; Figures 3E-T; Supplementary Figure 4). As expected, a 4-day pattern was also absent in OVX and OVX+E2 animals (*p*>0.05 in both cases). Note that 5-daycycles occurred rarely in Intact rats and using 5-day bins rather than 4-day bins abolished significant differences by day of cycle for all groups (*data not shown*).

### CBT Increased in Pre-to-Mid Adolescence

As growth and metabolic rate increase in adolescence, we hypothesized that CBT would also increase during the period of most rapid growth, pre to mid puberty. Pre to mid puberty (p26–p41) was associated with a significant positive trend in CBT in Intact (*p*=5.75*10^−5^) and Intact+C animals prior to contraceptive administration (*p*=1.20*10^−4^; Figures 4A,B). Notably, implantation of the silastic capsule in OVX and OVX+E2 animals resulted in a transient (1day) increase in CBT (OVX *p*=0.01, OVX+E2 *p*=0.03; Figures 4C,D; Supplementary Figure 5). This surgical-recovery-associated rise was highly variable and did not differ between OVX and OVX+E2 animals (*p*=0.65; Supplementary Figure 5). Interestingly, the early pubertal CBT increase did not require E2, as OVX animals also exhibited a significant positive trend (*p*=0.034; Figures 4C,E). For a summary guide to CBT features that may be useful for Intact pubertal staging, see Supplementary Figure 6.

### CBT Maintenance in Late Adolescence to Adulthood Required Estradiol

Complex interactions exist between metabolism, growth, and E2 level during adolescence. As estrogen deficiency in puberty is associated with weight gain and reduced metabolic rate, we investigated if maintenance of elevated temperature would be impacted by hormonal status. The maintenance of increased temperature in late puberty and adulthood was E2-dependent, with OVX animals exhibiting a significant downward trend in CBT from mid to late puberty (p58–p74; *p*=0.01) relative to Intact and OVX+E2 animals (*p*>0.05 in each case; Figures 4C,D). E2 treatment in the OVX+E2 group prevented intra-individual CBT decline in late puberty; correspondingly, temperatures in the OVX but not OVX+E2 groups were lower than that of Intact animals (late puberty to early adulthood *χ*^2^=62.8, *p*=1.46*10^--13^, *p*=4*10^−4^ for Intact vs. OVX, *p*=0.16 for Intact vs. OVX+E2; Figures 4F,G; Supplementary Figure 5).

### Contraceptive Administration Longitudinally Depressed CBT

Core body temperature power did not exhibit a positive or negative trend from mid puberty through early adulthood (p42–p58) in Intact animals (*p*=0.12), but exhibited a significant downward trend in Intact+C animals during the period of contraceptive administration (*p*=0.028; Figures 4A,B), resulting in a trend toward depressed temperatures following administration in mid to late adolescence (*χ*^2^=21.84, *p*=7.04*10^−05^, *p*=0.1 for Intact vs. Intact+C) that persisted into early adulthood (*χ*^2^=62.83, *p*=1.46*10^−13^, *p*=0.1 for Intact vs. Intact+C; Figures 4B,F,G).

## Discussion

### Adolescent Development and Estradiol Dependence of CBT Rhythmicity

The present findings reveal that female adolescence is characterized by stereotyped development of CBT rhythms at the ultradian, circadian, and ovulatory timescales. Early adolescence in the female rat is marked by rising CBT and CBT circadian power, and commencement of 4-day cycling in CBT and CBT URs. These early circadian and ultradian changes likely reflect maturation of the reproductive axis, as (1) the rate of CR power rise was hastened by estradiol, (2) the commencement of 4-day temperature cycling was preceded by a rise in fE2, and (3) CBT CR power was correlated with fE2 concentration (Delemarre-Van De Waal et al., 1991; Apter et al., 1993). These observations confirm and extend reports of early pubertal development of ultradian-circadian-ovulatory interactions within the hypothalamus (Mohr et al., 2017, 2019), and suggest that reproductive-thermoregulatory coupling may develop prior to, or in tandem with, pubertal onset.

Adolescent increases in UR amplitude for many endocrine outputs have been reported at discreet points in time (e.g., Tanner Stage 1 vs. 2), but continuous changes to UR structure across all of puberty, or in peripheral markers, are not well-mapped (Delemarre-Van De Waal et al., 1991; Dunger et al., 1991a). We found that, from the time of rise in fE2 (a defining marker of pubertal onset), CBT UR power retained the same mean value but commenced a 4-day, ovulatory cycle-associated pattern. Four day cycles in URs are consistent with data collected on URs in adult rodents (Sanchez-Alavez et al., 2011; Prendergast et al., 2012; Smarr et al., 2017) and in women (at a longer time scale; Grant et al., 2020a). Four-day patterning was not present in ovariectomized or E2-replaced animals, consistent with dependence on the ovarian cycle. These results reveal that URs in CBT achieve adult amplitude and stability early in life, prior to maturity of CRs (Tenreiro et al., 1991; Menna-Barreto et al., 1993; Bueno and Menna-Barreto, 2016), and that interactions among thermoregulatory and reproductive circuits permitting rapid *ultradian* coupling are established well before pubertal onset.

Additionally, the present findings suggest that some dynamics of CBT development require specific patterns of E2 rather than simply concentrations above a particular threshold. Ovariectomy eliminated ORs, increased weight, reduced CR power, rate of CR power rise, and correlation between CR power and fE2, and overall temperature (Wegorzewska et al., 2008; Curtis et al., 2018). E2 replacement partially rescued circadian metrics and reduced weight but did not recapitulate ORs. Short-term exposure to contraceptives in late adolescence longitudinally altered CBT metrics by reducing temperature and CR power, and abolishing ORs in CBT and UR power. Despite the apparently early maturation of substrate for thermoregulatory and reproductive coupling, the impact of E2 replacement and the enduring effects of short-term contraceptives suggest that these systems are sensitive to both level and patterning of reproductive hormones across the adolescent period.

Together, temperature amplitude and oscillation stability at the UR, CR, and OR timescales in the present study are modulated across the adolescent transition and are influenced by endogenous and exogenous E2. CBT URs mature to their adult amplitude and stability prior to CRs, with the dominant early pubertal change manifesting as a modulation of amplitude and stability by ovulatory phase in intact animals (See Supplementary Figure 6). Conversely, CRs increase in magnitude and stability in early adolescence, are not significantly impacted by the phase of OR, and are impacted by ovariectomy. These results support the notion that ultradian and ovulatory systems are tightly coupled and reflected in CBT, and that circadian effects on the ovulatory cycle may be unidirectional (Shechter and Boivin, 2010; Grant et al., 2020a). Ovariectomy, E2 replacement, and pubertal contraceptive administration have a number of effects on rhythmic dynamics at each timescale, overall indicating that “intact” dynamics are not easily recapitulated and that exogenous sex steroids can have enduring impact (See Supplementary Figure 7).

### Limitations

The ovulatory cycle of the female rat differs from that of humans, in that rats do not exhibit prolonged elevation of progesterone in the absence of pregnancy or pseudopregnancy (Paccola et al., 2013). This lack of a true luteal phase means that post-ovulatory temperature elevation in the rat follows a more compressed trajectory than in humans (Marrone et al., 1976). Therefore, exposure to estrogen and progesterone analogs in rats for prolonged periods may be associated with a different phenotype in rats than in humans (Liechty et al., 2015). Additionally, the present study focused on CBT, as opposed to locomotor activity (LA). LA is known to exhibit rhythms closely related to those of CBT during the active phase, and LA CRs are known exhibit analogous increases in amplitude and regularity from pre to mid puberty in intact males (Hagenauer et al., 2011). Finally, the laboratory environment imposes artificially stable environmental conditions on animals; recapitulation of these patterns under naturalistic conditions in future experiments will strengthen the translational potential of this work.

### Considerations of Rhythmicity Perturbation Through Adolescent Contraceptive Use

Although rats cannot fully model human biology, it was notable that contraceptive administration imposed lasting structural changes on CBT rhythmicity and its relationship with E2. Levonorgestrel and EE2 administration abolished the 4-day modulation of fE2, UR power, and temperature level, eliminated the correlation between CR power and fE2, and significantly depressed CR power. As these changes required continuous monitoring to detect and occurred in the absence of significant group changes to fE2 level, it is not unreasonable to speculate that studies of less frequently timed samples, or samples averaged across individuals, could make similar disruptions in humans difficult to detect. Whether or not contraceptive administration at other times of adolescence, or in adulthood, would generate a different phenotype than that observed here represents an important area of further inquiry.

Perturbation of body temperature rhythms is associated with diverse health insults across species, both reflecting perturbation in underlying systems and potentially acting as a causative agent. Circadian disruption to CBT rhythms occurs in, and is proportional to, severity of jetlag (Ariznavarreta et al., 2002), depression (Polugrudov et al., 2016), sepsis severity (Drewry et al., 2013; Granger et al., 2013), post-traumatic injury (Culver et al., 2020), cognitive decline (Sharma et al., 2021), and has even been proposed as a root cause of disease dubbed “Circadian Syndrome” (Zimmet et al., 2019). Disruption to ovulatory temperature rhythms occurs in anovulatory and atypical luteal phase cycles (Akin and Elstein, 1975; Bopp and Shoupe, 1993; He, 1993), including those arising from polycystic ovarian syndrome (PCOS; Mitwally et al., 1999). The impact of ultradian rhythmic disruption of the reproductive axis requires additional study (Veldhuis et al., 1988; Sir-Petermann et al., 1999; Herbison, 2018), but existing work in other hormonal systems suggests that preservation of pulsatility in drug delivery (e.g., of cortisol in Addison’s disease or insulin in diabetes) can lead to better patient outcomes when compared to conventional non-rhythmic treatment (Lightman and Terry, 2014; Smarr et al., 2016a; Choi et al., 2018; Kalafatakis et al., 2018; Lightman et al., 2020). It is likely that disruption of URs may result in negative impact analogous to disruption at longer timescales. It may appear counter-intuitive that thermoregulation could be both a reporter for such diverse maladies and a potential mediator for disease progression. However, temperature rhythm disruption is associated with a wide range of temporal, inflammatory, and endocrine insults, in part, because thermoregulatory circuits are directly impacted by the master clock, and modulated by inflammatory factors, autonomic status, and a variety of endocrine factors including estradiol (Williams et al., 2010; Marui et al., 2016) and progesterone (Forman et al., 1987). Lastly, CBT itself acts as a synchronizing cue for peripheral circadian oscillators (Brown et al., 2002). Further research is needed to disentangle if disruption of CBT rhythmicity itself causes harm, or if CBT rhythms are merely reporting perturbations in underlying systems (e.g., sex hormones or metabolic factors).

Our observations suggest that contraceptive administration that results in a non-physiological temporal endocrine pattern may act as “hormonal jetlag.” This evidence is consistent with previous evidence that hormonal contraceptive use is associated with elevated body temperature (Baker et al., 2001), decoupling of follicular maturation cycles within the ovary (Obruca et al., 2001; Landersoe et al., 2020), weight change (Lopez et al., 2016; Okunola et al., 2019), mental health risks (Skovlund et al., 2016, 2018; Fruzzetti and Fidecicchi, 2020), lasting luteal phase deficiency (Gnoth et al., 2002), and a variety of other off target effects (Barr, 2010; Benagiano et al., 2019). Furthermore, women under 21 are more likely to exhibit anovulatory cycles following birth control cessation than are older individuals (Pinkerton and Carey, 1976), suggesting that contraceptives taken during late adolescence may be more disruptive than in adulthood.

The degree of lasting impact to other systems that rely on temperature as an entraining stimulus, or disrupted systems reported indirectly by temperature (e.g., SCN, endocrine, and autonomic) remain to be assessed. Furthermore, the extent to which such disruptive effects differ among species (Liechty et al., 2015), contraceptive agents, administration methods, or between adolescent populations and adults requires further investigation. Encouragingly, the observation that rodents and humans exhibit similar CBT patterning during the peri-ovulatory period (Sanchez-Alavez et al., 2010; Smarr et al., 2017; Grant et al., 2020a) points to potential translational relevance.

Together, despite established societal benefits of widely available hormonal contraception (Peachman, 2018), especially in individuals experiencing hormonal irregularities (Bahamondes et al., 2015), the present findings suggest that administration of exogenous estrogens and progestins during adolescence leads to persistent rhythmic disruption across timescales. Future research is needed to determine if rhythmic patterns of sex steroid administration more closely mimicking endogenous release, analogous to those implemented in cortisol (Kalafatakis et al., 2018) and closed loop insulin therapy (Lewis, 2019), can minimize rhythmic disruption. Conversely, future studies that validate an “updated” symptom-thermal method using signal processing of continuous CBT data may provide feasible, non-disruptive alternatives for contraception (Aptekar et al., 2016; Shilaih et al., 2017; Grant et al., 2020a; Webster and Smarr, 2020). Contraceptive administration to adolescent girls is on the rise (Birth Control Pill Use-Child Trends, 2018), and there are a paucity of data on the impact of chronic hormonal perturbation on endogenous rhythmicity, or if such disruptions during the sensitive window of adolescence have lasting effects (Pinkerton and Carey, 1976; Skovlund et al., 2016, 2018; Mørch et al., 2017; Westhoff and Pike, 2018). Further research is needed to characterize the impact of adolescent hormonal contraceptive use so that further improvements can be made, individuals at-risk for side effects can be identified, and informed decisions about family planning can be made.

### Utility of CBT for Monitoring Pubertal Development in Rodents and Potential Translational Relevance

Continuous monitoring of CBT may have great utility for passive detection of pubertal milestones in rodents in preclinical research. Existing methods for pubertal staging in rodents carry considerable downsides: frequent blood sampling and vaginal lavage (McLean et al., 2012) are repeatedly invasive, and fecal hormone analysis is time consuming and costly (Chelini et al., 2005; Woodruff et al., 2010). Moreover, our results indicate that individual rats do not traverse identical pubertal trajectories by day of life, and that this assumption could lead to considerable errors in staging. Conversely, signal processing of passively collected CBT can add temporal resolution, greater quantitative power, and limit repeated invasive procedures for staging puberty (van der Vinne et al., 2020).

In human subjects, continuous temperature monitoring *via* wearables may similarly facilitate the study of pubertal development. The signal characteristics of rat CBT exhibit remarkable similarities to human peripheral temperature (See: Sanchez-Alavez et al., 2011; Shechter et al., 2011; Grant et al., 2020b). Monitoring human peripheral temperature during adolescence could serve broad purposes – from personalizing health education based on self-collected data (Eschler et al., 2019; Fowler et al., 2020), to adapting teaching style to an individual’s developmental phase (Davidow et al., 2016; DePasque and Galván, 2017; Piekarski et al., 2017; Master et al., 2020), to enabling research into the impact of teen contraceptive use (Martinez, 2015; Patseadou and Michala, 2017) or the process of gender transition (Castilla-Peón, 2018; Strittmatter and Holtmann, 2020). Together, pubertal monitoring *via* continuous body temperature is worthy of further investigation in both animal models and human subject populations for its potential utility to individuals, researchers, families, and clinicians.

## Conclusion

In conclusion, body temperature monitoring provides a window into the development of biological rhythms in puberty over multiple timescales. These rhythms may serve as convenient and high temporal resolution indicators of developmental stage and trajectory for application in research and clinical studies. Our study of body temperature also reveals unintended side effects of tonic hormonal manipulations. We anticipate that these findings will inform creative improvements to female reproductive research and healthcare.

## Data Availability Statement

The raw data supporting the conclusions of this article will be made available by the authors, without undue reservation. All code and data used in this paper are available at A.G.’s and L.J.K.’s Github Repository.

## Author Contributions

ADG, LW, and LJK: conceptualization, methodology, data interpretation and analysis, writing – original draft, and writing – review and editing. ADG: investigation. LW and LJK: funding acquisition, resources, and supervision. All authors contributed to the article and approved the submitted version.

## Funding

This work was funded by SL-CN grant #1640885 (LW) and NIH grant HD-050470 (LJK).

## Conflict of Interest

Following completion of this study and her PhD, and following the original submission of this manuscript, AG accepted a position at Prima-Temp, a company that applies a core temperature-monitoring cervical ring to monitor female fertility and health.

The remaining authors declare that the research was conducted in the absence of any commercial or financial relationships that could be construed as a potential conflict of interest.

## Publisher’s Note

All claims expressed in this article are solely those of the authors and do not necessarily represent those of their affiliated organizations, or those of the publisher, the editors and the reviewers. Any product that may be evaluated in this article, or claim that may be made by its manufacturer, is not guaranteed or endorsed by the publisher.

## Footnotes

^1^https://github.com/azuredominique; https://github.com/Kriegsfeld-Lab

## Supplementary Material

Supplementary Figure 1 Fecal estradiol (fE2) group averages from pre-puberty to adulthood post-contraception: contraceptives longitudinally alter the pattern but not level of fecal estradiol (fE2). Pre-puberty (p24–p30), early puberty (p30–p37), mid puberty (p43–p49), late puberty (p55–p61), and early adulthood (p69–p76) in Intact **(A)**, Intact+C **(B)**, OVX **(C)**, and OVX+E2 **(D)**. Early adulthood post-contraceptive administration, captured only in Intact and Intact+C animals, are overlaid in **(E)**, illustrating persistent perturbation of estrous-cycle fE2 patterning. Dark bar indicates samples were collected during contraceptive administration in Intact+C animals. * indicates significant elevation of day 3 compared to day 1 (p<0.05).

Supplementary Figure 2 Ovariectomized (OVX) and OVX+E2 are heavier than Intact animals in mid puberty and early adulthood. Prepubertal OVX did not significantly impact weight gain before mid-puberty. OVX animals (gray) are significantly heavier than all other groups at pre to mid-puberty. OVX animals remain significantly heavier into adulthood. In early adulthood, OVX+E2 animals (orange) are not different from OVX animals and are significantly heavier than either Intact (light blue) or Intact+C animals (dark blue). Colors of * indicate groups that are significantly heavier (p<0.05).

Supplementary Figure 3 Day of first estradiol rise occurs near vaginal opening and coincides with markers of estrus: subsequent drop in ultradian rhythm (UR) power and rise in core body temperature (CBT). Estradiol has a unique day in proximity to, but distinct from, vaginal opening, during which fE2 rises >2 SDs within an individual **(A,B)**. Alignment to this day as a proxy for puberty onset provides a convenient point from which to average across animals to permit easy visualization of the estrous cycle. Daily mean UR power decreases significantly following this day **(C)**, and daily mean CBT rises significantly following this day **(D)**. The difference between these two metrics exaggerates the difference and the differences across subsequent cycles **(E)**. After this point, all three metrics commence the 4-day estrous pattern. * indicates significant difference from all other time periods.

Supplementary Figure 4 Fast Fourier Transform (FFT) power of Intact temperature across life peaks unique at 4days and is disrupted during and after contraceptive administration. Fast Fourier Transform of each individual’s CBT log in Intact (light blue) and Intact+BC (dark blue) prior to contraceptive administration **(left)**, and during and after contraceptive administration **(right)**. * indicates significant difference for AUC between the groups in 4–5 day periodicity.

Supplementary Figure 5 Estradiol administration in ovariectomized animals is associated with lower temperatures across adolescence. Core CBT linear group means (±SD) in OVX (gray) and OVX+E2 animals from p28 to p72. Box plots indicate values used in Kruskal-Wallis (KW) comparisons between groups during silastic implant recovery **(left)** and across adolescence **(right)** with independent y-axes. * indicates significant difference between groups during the marked time period (p<0.03).

Supplementary Figure 6 Summary of CBT feature use for pubertal staging in an Intact female. Features of CBT and CBT rhythmicity can be used to complement and extend hormonal and external markers of adolescence. Here, we illustrate derived features on an example Intact female from p26 to p74, with fE2 rise and vaginal opening occurring on p31 and p32, respectively. Dots indicate vaginal opening and “e” indicates first rise of fE2 >2 SDs. Rising CBT, emergence of ovulatory rhythms (ORs) in UR power, and rising circadian rhythm (CR) power can be used to characterize pubertal onset. Mid to late adolescence is characterized by a plateau of CR power, as well as the emergence of ORs in raw CBT their persistence in UR power. Post-natal day 60 did not correspond with any overt transitions in CBT or CBT metrics.

Supplementary Figure 7 Examples of individual fE2 and CBT. Representative individual plots of fE2 (top) and CBT (bottom) from Intact **(A)**, Intact+C **(B)**, OVX **(C)**, and OVX+E2 animals. Gray bars indicate windows of time during which fE2 was not collected. Horizontal bar in B indicates days during which hormonal contraception was administered.

## References

[ref1] Adeyemi-Fowode O. A. Santos X. M. Dietrich J. E. Srivaths L. (2017). Levonorgestrel-releasing intrauterine device use in female adolescents with heavy menstrual bleeding and bleeding disorders: single institution review. J. Pediatr. Adolesc. Gynecol. 30, 479–483. doi: 10.1016/j.jpag.2016.04.001, PMID: 27108228

[ref2] Akin A. Elstein M. (1975). The value of the basal temperature chart in the management of infertility. Int. J. Fertil. 20, 122–124. PMID: 3476

[ref3] Albertsson-Wikland K. Rosberg S. Lannering B. Dunkel L. Selstam G. Norjavaara E. (1997). Twenty-four-hour profiles of luteinizing hormone, follicle-stimulating hormone, testosterone, and estradiol levels: a semilongitudinal study throughout puberty in healthy boys. J. Clin. Endocrinol. Metab. 82, 541–549. doi: 10.1210/jcem.82.2.3778, PMID: 9024251

[ref4] Andrews W. W. Advis J. P. Ojeda S. R. (1981). The maturation of estradiol-negative feedback in female rats: evidence that the resetting of the hypothalamic “gonadostat” does not precede the first preovulatory surge of gonadotropins. Endocrinology 109, 2022–2031. doi: 10.1210/endo-109-6-2022, PMID: 6796387

[ref5] Angelopoulou E. Quignon C. Kriegsfeld L. J. Simonneaux V. (2019). Functional implications of RFRP-3 in the central control of daily and seasonal rhythms in reproduction. Front. Endocrinol. 10:183. doi: 10.3389/fendo.2019.00183, PMID: 31024442 PMC6467943

[ref6] Aptekar D. Costantini L. Katilius J. Webster W. (2016). Continuous, passive personal wearable sensor to predict ovulation [21G]. Obstet. Gynecol. 127:64S. doi: 10.1097/01.AOG.0000483905.29999.b1

[ref7] Apter D. (1997). Development of the hypothalamic-pituitary-ovarian axis. Ann. N. Y. Acad. Sci. 816, 9–21. doi: 10.1111/j.1749-6632.1997.tb52125.x, PMID: 9238251

[ref8] Apter D. (2018). Contraception options: aspects unique to adolescent and young adult. Best Pract. Res. Clin. Obstet. Gynaecol. 48, 115–127. doi: 10.1016/j.bpobgyn.2017.09.010, PMID: 29032945

[ref9] Apter D. Bützow T. L. Laughlin G. A. Yen S. S. (1993). Gonadotropin-releasing hormone pulse generator activity during pubertal transition in girls: pulsatile and diurnal patterns of circulating gonadotropins. J. Clin. Endocrinol. Metab. 76, 940–949. doi: 10.1210/jcem.76.4.8473410, PMID: 8473410

[ref10] Apter D. Bützow T. Laughlin G. A. Yen S. S. (1994). Accelerated 24-hour luteinizing hormone pulsatile activity in adolescent girls with ovarian hyperandrogenism: relevance to the developmental phase of polycystic ovarian syndrome. J. Clin. Endocrinol. Metab. 79, 119–125. doi: 10.1210/jcem.79.1.8027216, PMID: 8027216

[ref11] Ariznavarreta C. Cardinali D. P. Villanúa M. A. Granados B. Martín M. Chiesa J. J.. (2002). Circadian rhythms in airline pilots submitted to long-haul transmeridian flights. Aviat. Space Environ. Med. 73, 445–455. PMID: 12014603

[ref13] Auer K. E. Kußmaul M. Möstl E. Hohlbaum K. Rülicke T. Palme R. (2020). Measurement of fecal testosterone metabolites in mice: replacement of invasive techniques. Animals 10:165. doi: 10.3390/ani10010165, PMID: 31963733 PMC7023058

[ref14] azuredominique (2021). azuredominique/Rat-Puberty-Lab-Conditions. Available at: https://github.com/azuredominique/Rat-Puberty-Lab-Conditions (Accessed March 3, 2021).

[ref15] Backstrom C. McNeilly A. S. Leask R. Baird D. (1982). Pulsatile secretion of LH, FSH, prolactin, oestradiol, and progesterone during the human menstrual cycle. Clin. Endocrinol. 17, 29–42. doi: 10.1111/j.1365-2265.1982.tb02631.x, PMID: 6811166

[ref16] Bahamondes L. Bahamondes M. V. Shulman L. P. (2015). Non-contraceptive benefits of hormonal and intrauterine reversible contraceptive methods. Hum. Reprod. Update 21, 640–651. doi: 10.1093/humupd/dmv023, PMID: 26037216

[ref17] Baker F. C. Mitchell D. Driver H. S. (2001). Oral contraceptives alter sleep and raise body temperature in young women. Pflugers Arch. 442, 729–737. doi: 10.1007/s004240100582, PMID: 11512029

[ref18] Bakshi V. P. Geyer M. A. (1999). Ontogeny of isolation rearing-induced deficits in sensorimotor gating in rats. Physiol. Behav. 67, 385–392. doi: 10.1016/S0031-9384(99)00082-7, PMID: 10497957

[ref19] Barr N. G. (2010). Managing adverse effects of hormonal contraceptives. Am. Fam. Physician 82, 1499–1506. PMID: 21166370

[ref20] Benagiano G. Benagiano M. Bianchi P. D’Elios M. M. Brosens I. (2019). Contraception in autoimmune diseases. Best Pract. Res. Clin. Obstet. Gynaecol. 60, 111–123. doi: 10.1016/j.bpobgyn.2019.05.003, PMID: 31160225

[ref21] Birth Control Pill Use-Child Trends (2018). Available at: https://www.childtrends.org/indicators/birth-control-pill-use (Accessed January 8, 2021).

[ref22] Blum I. D. Zhu L. Moquin L. Kokoeva M. V. Gratton A. Giros B.. (2014). A highly tunable dopaminergic oscillator generates ultradian rhythms of behavioral arousal. Elife 3:e05105. doi: 10.7554/eLife.05105, PMID: 25546305 PMC4337656

[ref23] Boggiano M. M. Cavigelli S. A. Dorsey J. R. Kelley C. E. P. Ragan C. M. Chandler-Laney P. C. (2008). Effect of a cage divider permitting social stimuli on stress and food intake in rats. Physiol. Behav. 95, 222–228. doi: 10.1016/j.physbeh.2008.04.025, PMID: 18565550 PMC2562762

[ref24] Bopp B. Shoupe D. (1993). Luteal phase defects. J. Reprod. Med. 38, 348–356. PMID: 8320670

[ref25] Bourguignon J. P. (1988). Time-related neuroendocrine manifestations of puberty: a combined clinical and experimental approach extracted from the 4th Belgian endocrine society lecture. Horm. Res. 30, 224–234. doi: 10.1159/000181068, PMID: 3150758

[ref26] Brainard G. C. (1988). “Illumination of Animals in Microgravity Habitats,” in NASA Reports.

[ref27] Brandenberger G. Simon C. Follenius M. (1987). Ultradian endocrine rhythms: a multioscillatory system. J. Interdiscip. Cycle Res. 18, 307–315. doi: 10.1080/09291018709359958

[ref29] Brodsky V. Y. (2014). Circahoralian (Ultradian) metabolic rhythms. Biochemistry 79, 483–495. doi: 10.1134/S0006297914060017, PMID: 25100006

[ref30] Brodsky V. Y. Lloyd D. (2008). “Self-organized intracellular ultradian rhythms provide direct cell-cell communication,” in Ultradian Rhythms from Molecules to Mind: A New Vision of Life. eds. Lloyd D. Rossi E. L. (Dordrecht: Springer Netherlands), 85–104.

[ref31] Brown S. A. Zumbrunn G. Fleury-Olela F. Preitner N. Schibler U. (2002). Rhythms of mammalian body temperature can sustain peripheral circadian clocks. Curr. Biol. 12, 1574–1583. doi: 10.1016/S0960-9822(02)01145-4, PMID: 12372249

[ref32] Bueno C. Menna-Barreto L. (2016). Development of sleep/wake, activity and temperature rhythms in newborns maintained in a neonatal intensive care unit and the impact of feeding schedules. Infant Behav. Dev. 44, 21–28. doi: 10.1016/j.infbeh.2016.05.004, PMID: 27261553

[ref34] Carlson L. J. Shaw N. D. (2019). Development of ovulatory menstrual cycles in adolescent girls. J. Pediatr. Adolesc. Gynecol. 32, 249–253. doi: 10.1016/j.jpag.2019.02.119, PMID: 30772499 PMC6570576

[ref35] Carskadon M. A. Harvey K. Duke P. Anders T. F. Litt I. F. Dement W. C. (2002). Pubertal changes in daytime sleepiness. 1980. Sleep 25, 453–460. PMID: 12224838

[ref36] Casper R. F. Gladanac B. (2014). Introduction: circadian rhythm and its disruption: impact on reproductive function. Fertil. Steril. 102, 319–320. doi: 10.1016/j.fertnstert.2014.04.053, PMID: 24954773

[ref37] Castilla-Peón M. F. (2018). Medical management of transgender children and adolescents. Bol. Med. Hosp. Infant. Mex. 75, 7–14. doi: 10.24875/BMHIM.M18000003, PMID: 29652872

[ref38] Chelini M. O. M. Souza N. L. Rocha A. M. Felippe E. C. G. Oliveira C. A. (2005). Quantification of fecal estradiol and progesterone metabolites in Syrian hamsters (Mesocricetus auratus). Braz. J. Med. Biol. Res. 38, 1711–1717. doi: 10.1590/S0100-879X2005001100021, PMID: 16258643

[ref39] Choi S. B. Hong E. S. Noh Y. H. (2018). Open Artificial Pancreas System Reduced Hypoglycemia and Improved Glycemic Control in Patients with Type 1 Diabetes|Diabetes. Available at: https://diabetes.diabetesjournals.org/content/67/Supplement_1/964-P (Accessed September 10, 2020).

[ref40] Clark R. G. Tarttelin M. F. (1982). Some effects of ovariectomy and estrogen replacement on body composition in the rat. Physiol. Behav. 28, 963–969. doi: 10.1016/0031-9384(82)90161-5, PMID: 7111460

[ref42] Coukell A. J. Balfour J. A. (1998). Levonorgestrel subdermal implants. A review of contraceptive efficacy and acceptability. Drugs 55, 861–887. doi: 10.2165/00003495-199855060-00019, PMID: 9617600

[ref43] Crowley S. J. Cain S. W. Burns A. C. Acebo C. Carskadon M. A. (2015). Increased sensitivity of the circadian system to light in early/mid-puberty. J. Clin. Endocrinol. Metab. 100, 4067–4073. doi: 10.1210/jc.2015-2775, PMID: 26301944 PMC4702443

[ref44] Culver A. Coiffard B. Antonini F. Duclos G. Hammad E. Vigne C.. (2020). Circadian disruption of core body temperature in trauma patients: a single-center retrospective observational study. J. Intensive Care 8:4. doi: 10.1186/s40560-019-0425-x, PMID: 31921428 PMC6945723

[ref45] Curtis K. S. McCracken K. Espinosa E. Ong J. Buck D. J. Davis R. L. (2018). Temporal and site-specific changes in central neuroimmune factors during rapid weight gain after ovariectomy in rats. Neurochem. Res. 43, 1802–1813. doi: 10.1007/s11064-018-2596-6, PMID: 30030770

[ref46] Davidow J. Y. Foerde K. Galván A. Shohamy D. (2016). An upside to reward sensitivity: the hippocampus supports enhanced reinforcement learning in adolescence. Neuron 92, 93–99. doi: 10.1016/j.neuron.2016.08.031, PMID: 27710793

[ref47] Day M. L. Imakawa K. Garcia-Winder M. Kittok R. J. Schanbacher B. D. Kinder J. E. (1986). Influence of prepubertal ovariectomy and estradiol replacement therapy on secretion of luteinizing hormone before and after pubertal age in heifers. Domest. Anim. Endocrinol. 3, 17–25. doi: 10.1016/0739-7240(86)90036-6

[ref48] de Kloet E. R. Sarabdjitsingh R. A. (2008). Everything has rhythm: focus on glucocorticoid pulsatility. Endocrinology 149, 3241–3243. doi: 10.1210/en.2008-0471, PMID: 18586914

[ref49] Delemarre-Van De Waal H. A. Wennink J. M. Odink R. J. (1991). Gonadotrophin and growth hormone secretion throughout puberty. Acta Paediatr. Scand. Suppl. 372, 26–31. doi: 10.1111/j.1651-2227.1991.tb17964.x, PMID: 1927517

[ref50] DePasque S. Galván A. (2017). Frontostriatal development and probabilistic reinforcement learning during adolescence. Neurobiol. Learn. Mem. 143, 1–7. doi: 10.1016/j.nlm.2017.04.009, PMID: 28450078

[ref51] Dibner C. Schibler U. Albrecht U. (2010). The mammalian circadian timing system: organization and coordination of central and peripheral clocks. Annu. Rev. Physiol. 72, 517–549. doi: 10.1146/annurev-physiol-021909-135821, PMID: 20148687

[ref52] Drewry A. M. Fuller B. M. Bailey T. C. Hotchkiss R. S. (2013). Body temperature patterns as a predictor of hospital-acquired sepsis in afebrile adult intensive care unit patients: a case-control study. Crit. Care 17:R200. doi: 10.1186/cc12894, PMID: 24028682 PMC3906745

[ref53] Duan X. N. Yan S. Q. Wang S. M. Hu J. J. Fang J. Gong C.. (2018). Developmental characteristics of circadian rhythms in hypothalamic-pituitary-adrenal axis during puberty. Zhonghua Liu Xing Bing Xue Za Zhi 39, 1086–1090. doi: 10.3760/cma.j.issn.0254-6450.2018.08.014, PMID: 30180433

[ref54] Dunger D. B. Matthews D. R. Edge J. A. Jones J. Preece M. A. (1991a). Evidence for temporal coupling of growth hormone, prolactin, LH and FSH pulsatility overnight during normal puberty. J. Endocrinol. 130, 141–149. doi: 10.1677/joe.0.1300141, PMID: 1908888

[ref55] Dunger D. B. Villa A. K. Matthews D. R. Edge J. A. Jones J. Rothwell C.. (1991b). Pattern of secretion of bioactive and immunoreactive gonadotrophins in normal pubertal children. Clin. Endocrinol. 35, 267–275. doi: 10.1111/j.1365-2265.1991.tb03534.x, PMID: 1742886

[ref56] Eschler J. Menking A. Fox S. Backonja U. (2019). Defining menstrual literacy with the aim of evaluating mobile menstrual tracking applications. Comput. Inform. Nurs. 37, 638–646. doi: 10.1097/CIN.0000000000000559, PMID: 31524688

[ref57] Follesa P. Porcu P. Sogliano C. Cinus M. Biggio F. Mancuso L.. (2002). Changes in GABAA receptor gamma 2 subunit gene expression induced by long-term administration of oral contraceptives in rats. Neuropharmacology 42, 325–336. doi: 10.1016/S0028-3908(01)00187-3, PMID: 11897111

[ref58] Forman R. G. Chapman M. C. Steptoe P. C. (1987). The effect of endogenous progesterone on basal body temperature in stimulated ovarian cycles. Hum. Reprod. 2, 631–634. doi: 10.1093/oxfordjournals.humrep.a136605, PMID: 3125209

[ref59] Fowler L. R. Gillard C. Morain S. (2020). Teenage use of smartphone applications for menstrual cycle tracking. Pediatrics 145:e20192954. doi: 10.1542/peds.2019-2954, PMID: 32291347

[ref60] Franklin A. D. Waddell W. T. Behrns S. Goodrowe K. L. (2020). Estrous cyclicity and reproductive success are unaffected by translocation for the formation of new reproductive pairs in captive red wolves (Canis rufus). Zoo Biol. 39, 230–238. doi: 10.1002/zoo.21551, PMID: 32476169

[ref61] Fruzzetti F. Fidecicchi T. (2020). Hormonal contraception and depression: updated evidence and implications in clinical practice. Clin. Drug Investig. 40, 1097–1106. doi: 10.1007/s40261-020-00966-8, PMID: 32980990

[ref62] Gabriel S. M. Roncancio J. R. Ruiz N. S. (1992). Growth hormone pulsatility and the endocrine milieu during sexual maturation in male and female rats. Neuroendocrinology 56, 619–625. doi: 10.1159/000126284, PMID: 1488093

[ref63] Garcia J. Rosen G. Mahowald M. (2001). Circadian rhythms and circadian rhythm disorders in children and adolescents. Semin. Pediatr. Neurol. 8, 229–240. doi: 10.1053/spen.2001.29044, PMID: 11768785

[ref65] Geraghty D. P. Byrne K. B. McPherson G. A. Burcher E. (1990). Renal and myocardial adrenoceptors in steroid contraceptive-induced hypertension in rats. Clin. Exp. Pharmacol. Physiol. 17, 567–578. doi: 10.1111/j.1440-1681.1990.tb01357.x, PMID: 2170069

[ref66] Gibson E. M. Humber S. A. Jain S. Williams W. P. Zhao S. Bentley G. E.. (2008). Alterations in RFamide-related peptide expression are coordinated with the preovulatory luteinizing hormone surge. Endocrinology 149, 4958–4969. doi: 10.1210/en.2008-0316, PMID: 18566114 PMC2582915

[ref67] Gibson E. M. Wang C. Tjho S. Khattar N. Kriegsfeld L. J. (2010). Experimental “jet lag” inhibits adult neurogenesis and produces long-term cognitive deficits in female hamsters. PLoS One 5:e15267. doi: 10.1371/journal.pone.0015267, PMID: 21152025 PMC2995744

[ref68] Ginther O. J. Pinaffi F. L. V. Khan F. A. Duarte L. F. Beg M. A. (2013). Circadian influence on the preovulatory LH surge, ovulation, and prolactin concentrations in heifers. Theriogenology 79, 528–533. doi: 10.1016/j.theriogenology.2012.11.003, PMID: 23244766

[ref69] Gnoth C. Frank-Herrmann P. Schmoll A. Godehardt E. Freundl G. (2002). Cycle characteristics after discontinuation of oral contraceptives. Gynecol. Endocrinol. 16, 307–317. doi: 10.1080/713603100, PMID: 12396560

[ref70] Goh G. H. Maloney S. K. Mark P. J. Blache D. (2019). Episodic ultradian events—ultradian rhythms. Biology 8:15. doi: 10.3390/biology8010015, PMID: 30875767 PMC6466064

[ref71] Gotlieb N. Moeller J. Kriegsfeld L. J. (2018). Circadian control of neuroendocrine function: implications for health and disease. Curr. Opin. Physiol. 5, 133–140. doi: 10.1016/j.cophys.2018.11.001, PMID: 30957055 PMC6446932

[ref72] Granger J. I. Ratti P.-L. Datta S. C. Raymond R. M. Opp M. R. (2013). Sepsis-induced morbidity in mice: effects on body temperature, body weight, cage activity, social behavior and cytokines in brain. Psychoneuroendocrinology 38, 1047–1057. doi: 10.1016/j.psyneuen.2012.10.010, PMID: 23146654 PMC3707484

[ref73] Grant A. D. Newman M. Kriegsfeld L. J. (2020a). Ultradian rhythms in heart rate variability and distal body temperature anticipate onset of the luteinizing hormone surge. Sci. Rep. 10:20378. doi: 10.1038/s41598-020-76236-6, PMID: 33230235 PMC7683606

[ref74] Grant A. D. Newman M. Kriegsfeld L. J. (2020b). Ultradian rhythms in heart rate variability and distal body temperature anticipate the luteinizing hormone surge onset. bioRxiv [Preprint]. doi: 10.1101/2020.07.15.205450 PMC7683606 33230235

[ref75] Grant A. D. Wilsterman K. Smarr B. L. Kriegsfeld L. J. (2018). Evidence for a coupled oscillator model of endocrine ultradian rhythms. J. Biol. Rhythm. 33, 475–496. doi: 10.1177/0748730418791423, PMID: 30132387 PMC6425759

[ref76] Guerra M. D. O. Souza E. R. D. Peters V. M. (2002). Reproductive performance of female wistar rat, descendent of mothers treated with levonorgestrel during the lactation. Rev. Assoc. Méd. Bras. 48, 135–139. doi: 10.1590/S0104-42302002000200032, PMID: 12205530

[ref77] Gupta N. J. Khare A. (2020). Disruption in daily eating-fasting and activity-rest cycles in Indian adolescents attending school. PLoS One 15:e0227002. doi: 10.1371/journal.pone.0227002, PMID: 31923256 PMC6953840

[ref78] Hagenauer M. H. King A. F. Possidente B. McGinnis M. Y. Lumia A. R. Peckham E. M.. (2011). Changes in circadian rhythms during puberty in Rattus norvegicus: developmental time course and gonadal dependency. Horm. Behav. 60, 46–57. doi: 10.1016/j.yhbeh.2011.03.001, PMID: 21397604 PMC3112245

[ref79] Hagenauer M. H. Lee T. M. (2012). The neuroendocrine control of the circadian system: adolescent chronotype. Front. Neuroendocrinol. 33, 211–229. doi: 10.1016/j.yfrne.2012.04.003, PMID: 22634481 PMC4762453

[ref80] Harper J. M. Austad S. N. (2000). Fecal glucocorticoids: a noninvasive method of measuring adrenal activity in wild and captive rodents. Physiol. Biochem. Zool. 73, 12–22. doi: 10.1086/316721, PMID: 10685902

[ref81] Hastings M. H. Maywood E. S. Brancaccio M. (2018). Generation of circadian rhythms in the suprachiasmatic nucleus. Nat. Rev. Neurosci. 19, 453–469. doi: 10.1038/s41583-018-0026-z, PMID: 29934559

[ref82] He H. (1993). Diagnosis of basal body temperature, serum progesterone and endometrial biopsy for luteal phase defect. Zhonghua Fu Chan Ke Za Zhi 28, 122–123. PMID: 8344087

[ref83] Herbison A. E. (2018). The gonadotropin-releasing hormone pulse generator. Endocrinology 159, 3723–3736. doi: 10.1210/en.2018-00653, PMID: 30272161

[ref84] Hoeger K. M. Kolp L. A. Strobl F. J. Veldhuis J. D. (1999). Evaluation of LH secretory dynamics during the rat proestrous LH surge. Am. J. Phys. 276, R219–R225. doi: 10.1152/ajpregu.1999.276.1.R219, PMID: 9887198

[ref85] Hummer D. L. Lee T. M. (2016). Daily timing of the adolescent sleep phase: insights from a cross-species comparison. Neurosci. Biobehav. Rev. 70, 171–181. doi: 10.1016/j.neubiorev.2016.07.023, PMID: 27450579

[ref86] JADELLE (n.d.) (Levonorgestrel Implants) for Subdermal Use 35. Available at: http://www.accessdata.fda.gov/drugsatfda_docs/label/2016/020544s010lbl.pdf (Accessed September 29, 2019).

[ref87] Jenner M. R. Kelch R. P. Kaplan S. L. Grumbach M. M. (1972). Hormonal changes in puberty: IV. Plasma estradiol, LH, and FSH in prepubertal children, pubertal females, and in precocious puberty, premature thelarche, hypogonadism, and in a child with a feminizing ovarian tumor. J. Clin. Endocrinol. Metab. 34, 521–530. doi: 10.1210/jcem-34-3-521, PMID: 5011256

[ref88] Kalafatakis K. Russell G. M. Harmer C. J. Munafo M. R. Marchant N. Wilson A.. (2018). Ultradian rhythmicity of plasma cortisol is necessary for normal emotional and cognitive responses in man. Proc. Natl. Acad. Sci. U. S. A. 115, E4091–E4100. doi: 10.1073/pnas.1714239115, PMID: 29632168 PMC5924881

[ref89] Kim S. H. Lundgren J. A. Bhabhra R. Collins J. S. Patrie J. T. Solorzano C. M. B.. (2018). Progesterone-mediated inhibition of the GnRH pulse generator: differential sensitivity as a function of sleep status. J. Clin. Endocrinol. Metab. 103, 1112–1121. doi: 10.1210/jc.2017-02299, PMID: 29300925 PMC6283412

[ref90] Kottler M. L. Coussieu C. Valensi P. Levi F. Degrelle H. (1989). Ultradian, circadian and seasonal variations of plasma progesterone and LH concentrations during the luteal phase. Chronobiol. Int. 6, 267–277. doi: 10.3109/07420528909056928, PMID: 2805155

[ref91] Kriegsfeld L. J. Silver R. Gore A. C. Crews D. (2002). Vasoactive intestinal polypeptide contacts on gonadotropin-releasing hormone neurones increase following puberty in female rats. J. Neuroendocrinol. 14, 685–690. doi: 10.1046/j.1365-2826.2002.00818.x, PMID: 12213129 PMC3271841

[ref500] Kriegsfeld-Lab (2021). GitHub. Available at: https://github.com/Kriegsfeld-Lab444 (Accessed May 24, 2021).

[ref93] Landersoe S. K. Forman J. L. Petersen K. B. Larsen E. C. Nøhr B. Hvidman H. W.. (2020). Ovarian reserve markers in women using various hormonal contraceptives. Eur. J. Contracept. Reprod. Health Care 25, 65–71. doi: 10.1080/13625187.2019.1702158, PMID: 31852271

[ref94] Lehman M. N. He W. Coolen L. M. Levine J. E. Goodman R. L. (2019). Does the KNDy model for the control of gonadotropin-releasing hormone pulses apply to monkeys and humans? Semin. Reprod. Med. 37, 71–83. doi: 10.1055/s-0039-3400254, PMID: 31847027 PMC9097242

[ref95] Leise T. L. (2013). Wavelet analysis of circadian and ultradian behavioral rhythms. J. Circadian Rhythms 11:5. doi: 10.1186/1740-3391-11-5, PMID: 23816159 PMC3717080

[ref96] Leise T. L. (2015). “Chapter five—wavelet-based analysis of circadian behavioral rhythms,” in Methods in Enzymology Circadian Rhythms and Biological Clocks, Part A. ed. Sehgal A. (London, England: Academic Press), 95–119. 10.1016/bs.mie.2014.10.011 25662453

[ref97] Levonorgestrel And Ethinyl Estradiol (Oral Route) Description and Brand Names-Mayo Clinic (n.d.). Available at: https://www.mayoclinic.org/drugs-supplements/levonorgestrel-and-ethinyl-estradiol-oral-route/description/drg-20406441 (Accessed March 3, 2021).

[ref98] Lewis D. M. (2019). Automated Insulin Delivery. Available at: https://www.artificialpancreasbook.com/ (Accessed September 10, 2020).

[ref100] Liechty E. R. Bergin I. L. Bell J. D. (2015). Animal models of contraception: utility and limitations. Open Access J. Contracept. 6, 27–35. doi: 10.2147/OAJC.S58754, PMID: 29386922 PMC5683139

[ref101] Lightman S. L. Birnie M. T. Conway-Campbell B. L. (2020). Dynamics of ACTH and cortisol secretion and implications for disease. Endocr. Rev. 41, 470–490. doi: 10.1210/endrev/bnaa002, PMID: 32060528 PMC7240781

[ref102] Lightman S. Terry J. R. (2014). The importance of dynamic signalling for endocrine regulation and drug development: relevance for glucocorticoid hormones. Lancet Diabetes Endocrinol. 2, 593–599. doi: 10.1016/S2213-8587(13)70182-7, PMID: 24731665

[ref103] Lilly J. M. Olhede S. C. (2012). Generalized Morse wavelets as a superfamily of analytic wavelets. IEEE Trans. Signal Process. 60, 6036–6041. doi: 10.1109/TSP.2012.2210890

[ref104] Lloyd D. Stupfel M. (1991). The occurrence and functions of ultradian rhythms. Biol. Rev. Camb. Philos. Soc. 66, 275–299. doi: 10.1111/j.1469-185X.1991.tb01143.x, PMID: 1932467

[ref105] Logan R. W. Hasler B. P. Forbes E. E. Franzen P. L. Torregrossa M. M. Huang Y. H.. (2018). Impact of sleep and circadian rhythms on addiction vulnerability in adolescents. Biol. Psychiatry 83, 987–996. doi: 10.1016/j.biopsych.2017.11.035, PMID: 29373120 PMC5972052

[ref106] Lopez L. M. Ramesh S. Chen M. Edelman A. Otterness C. Trussell J.. (2016). Progestin-only contraceptives: effects on weight. Cochrane Database Syst. Rev. 2016:CD008815. doi: 10.1002/14651858.CD008815.pub4, PMID: 27567593 PMC5034734

[ref107] Lucaccioni L. Trevisani V. Marrozzini L. Bertoncelli N. Predieri B. Lugli L.. (2020). Endocrine-disrupting chemicals and their effects during female puberty: a review of current evidence. Int. J. Mol. Sci. 21:2078. doi: 10.3390/ijms21062078, PMID: 32197344 PMC7139481

[ref108] Lv X. He C. Huang C. Hua G. Chen X. Timm B. K.. (2020). Reprogramming of ovarian granulosa cells by YAP1 leads to development of high-grade cancer with mesenchymal lineage and serous features. Sci. Bull. 65, 1281–1296. doi: 10.1016/j.scib.2020.03.040 PMC8654108 34888112

[ref109] MacKinnon P. C. B. Puig-Duran E. Laynes R. (1978). Reflections on the attainment of puberty in the rat: have circadian signals a role to play in its onset? J. Reprod. Fertil. 52, 401–412. doi: 10.1530/jrf.0.0520401, PMID: 344875

[ref110] Marrone B. L. Gentry R. T. Wade G. N. (1976). Gonadal hormones and body temperature in rats: effects of estrous cycles, castration and steroid replacement. Physiol. Behav. 17, 419–425. doi: 10.1016/0031-9384(76)90101-3, PMID: 1034937

[ref111] Martinez G. M. (2015). Sexual activity, contraceptive use, and childbearing of teenagers aged 15–19 in the United States. NCHS Data Brief 23, 1–8. PMID: 26199985

[ref112] Marui S. Uchida Y. Nagashima K. (2016). Daily changes of body temperature and heart rate are modulated after estradiol depletion in female rats. Anat. Physiol. 6:197. doi: 10.4172/2161-0940.1000197

[ref113] Master S. L. Eckstein M. K. Gotlieb N. Dahl R. Wilbrecht L. Collins A. G. E. (2020). Distentangling the systems contributing to changes in learning during adolescence. Dev. Cogn. Neurosci. 41:100732. doi: 10.1016/j.dcn.2019.100732, PMID: 31826837 PMC6994540

[ref114] Mathew L. Gaikwad A. Gonzalez A. Nugent E. K. Smith J. A. (2017). Evaluation of active hexose correlated compound (AHCC) in combination with anticancer hormones in orthotopic breast cancer models. Integr. Cancer Ther. 16, 300–307. doi: 10.1177/1534735417704948, PMID: 28438054 PMC5759944

[ref115] McLean A. C. Valenzuela N. Fai S. Bennett S. A. L. (2012). Performing vaginal lavage, crystal violet staining, and vaginal cytological evaluation for mouse estrous cycle staging identification. J. Vis. Exp. 67:e4389. doi: 10.3791/4389, PMID: 23007862 PMC3490233

[ref116] Menna-Barreto L. Benedito-Silva A. A. Marques N. de Andrade M. M. Louzada F. (1993). Ultradian components of the sleep-wake cycle in babies. Chronobiol. Int. 10, 103–108. doi: 10.3109/07420529309059698, PMID: 8500186

[ref117] Merkley C. M. Coolen L. M. Goodman R. L. Lehman M. N. (2015). Evidence for changes in numbers of synaptic inputs onto KNDy and GnRH neurones during the preovulatory LH surge in the ewe. J. Neuroendocrinol. 27, 624–635. doi: 10.1111/jne.12293, PMID: 25976424 PMC4809364

[ref118] Merkley C. M. Porter K. L. Coolen L. M. Hileman S. M. Billings H. J. Drews S.. (2012). KNDy (kisspeptin/neurokinin B/dynorphin) neurons are activated during both pulsatile and surge secretion of LH in the ewe. Endocrinology 153, 5406–5414. doi: 10.1210/en.2012-1357, PMID: 22989631 PMC3473209

[ref119] Millspaugh J. J. Washburn B. E. (2003). Within-sample variation of fecal glucocorticoid measurements. Gen. Comp. Endocrinol. 132, 21–26. doi: 10.1016/S0016-6480(03)00061-3, PMID: 12765640

[ref120] Mitwally M. F. Kuscu N. K. Yalcinkaya T. M. (1999). High ovulatory rates with use of troglitazone in clomiphene-resistant women with polycystic ovary syndrome. Hum. Reprod. 14, 2700–2703. doi: 10.1093/humrep/14.11.2700, PMID: 10548604

[ref121] Moenter S. M. Caraty A. Locatelli A. Karsch F. J. (1991). Pattern of gonadotropin-releasing hormone (GnRH) secretion leading up to ovulation in the ewe: existence of a preovulatory GnRH surge. Endocrinology 129, 1175–1182. doi: 10.1210/endo-129-3-1175, PMID: 1874164

[ref122] Mohr M. A. DonCarlos L. L. Sisk C. L. (2017). Inhibiting production of new brain cells during puberty or adulthood blunts the hormonally induced surge of luteinizing hormone in female rats. eNeuro 4, ENEURO.0133–17.2017. doi: 10.1523/ENEURO.0133-17.2017, PMID: 29098175 PMC5666323

[ref123] Mohr M. A. Sisk C. L. (2013). Pubertally born neurons and glia are functionally integrated into limbic and hypothalamic circuits of the male Syrian hamster. Proc. Natl. Acad. Sci. U. S. A. 110, 4792–4797. doi: 10.1073/pnas.1219443110, PMID: 23460698 PMC3607016

[ref124] Mohr M. A. Wong A. M. Tomm R. J. Soma K. K. Micevych P. E. (2019). Pubertal development of estradiol-induced hypothalamic progesterone synthesis. Horm. Behav. 111, 110–113. doi: 10.1016/j.yhbeh.2018.12.007, PMID: 30552874 PMC6527482

[ref125] Mørch L. S. Skovlund C. W. Hannaford P. C. Iversen L. Fielding S. Lidegaard Ø. (2017). Contemporary hormonal contraception and the risk of breast cancer. N. Engl. J. Med. 377, 2228–2239. doi: 10.1056/NEJMoa1700732, PMID: 29211679

[ref126] Mwanthi M. Zaenglein A. L. (2018). Update in the management of acne in adolescence. Curr. Opin. Pediatr. 30, 492–498. doi: 10.1097/MOP.0000000000000649, PMID: 29846254

[ref127] Naqvi R. H. Mitra S. B. Saksena I. F. Lindberg M. C. (1984). Pharmacokinetics of levonorgestrel in the rat. Contraception 30, 81–88. doi: 10.1016/0010-7824(84)90081-7, PMID: 6434231

[ref128] Nowaczyk-Dura G. Czekaj P. (1998). Effects of ethinylestradiol and levonorgestrel on morphology, ultrastructure and histoenzymatic activity of rat kidney. Physiol. Res. 47, 241–251. PMID: 9803470

[ref129] Obruca A. Korver T. Huber J. Killick S. R. Landgren B. Struijs M. J. (2001). Ovarian function during and after treatment with the new progestagen Org 30659. Fertil. Steril. 76, 108–115. doi: 10.1016/S0015-0282(01)01824-6, PMID: 11438328

[ref130] Ojeda S. R. Skinner M. K. (2006). “Puberty in the rat,” in Knobil and Neill’s Physiology of Reproduction. 3rd Edn. Vol. 3. eds. J. D. Neill, J. R. G. Challis, D. M. Kretser, D. W. Pfaff, J. S. Richards, T. M. Plant, and P. M. Wassarman, 2061–2126.

[ref131] Ojeda S. R. Urbanski H. F. Ahmed C. E. (1986). “The onset of female puberty: studies in the rat,” in Recent Progress in Hormone Research: Proceedings of the 1985 Laurentian Hormone Conference. ed. Greep R. O. (Boston: Academic Press), 385–442. 10.1016/b978-0-12-571142-5.50013-6 3090657

[ref132] Okunola T. O. Bola-Oyebamiji S. B. Sowemimo O. (2019). Comparison of weight gain between levonorgestrel and etonogestrel implants after 12 months of insertion. Int. J. Gynaecol. Obstet. 147, 54–58. doi: 10.1002/ijgo.12901, PMID: 31265128

[ref133] Olaniyi K. S. Olatunji L. A. (2019). Oral ethinylestradiol-levonorgestrel attenuates cardiac glycogen and triglyceride accumulation in high fructose female rats by suppressing pyruvate dehydrogenase kinase-4. Naunyn. Schmiedebergs Arch. Pharmacol. 392, 89–101. doi: 10.1007/s00210-018-1568-3, PMID: 30276420

[ref134] Olatunji L. A. Olaniyi K. S. Usman T. O. Abolarinwa B. A. Achile C. J. Kim I.-K. (2017). Combined oral contraceptive and nitric oxide synthesis inhibition synergistically causes cardiac hypertrophy and exacerbates insulin resistance in female rats. Environ. Toxicol. Pharmacol. 52, 54–61. doi: 10.1016/j.etap.2017.03.012, PMID: 28376377

[ref135] Ootsuka Y. de Menezes R. C. Zaretsky D. V. Alimoradian A. Hunt J. Stefanidis A.. (2009). Brown adipose tissue thermogenesis heats brain and body as part of the brain-coordinated ultradian basic rest-activity cycle. Neuroscience 164, 849–861. doi: 10.1016/j.neuroscience.2009.08.013, PMID: 19679172 PMC2767384

[ref136] Paccola C. C. Resende C. G. Stumpp T. Miraglia S. M. Cipriano I. (2013). The rat estrous cycle revisited: a quantitative and qualitative analysis. Anim. Reprod. 10, 677–683.

[ref137] Panda S. (2016). Circadian physiology of metabolism. Science 354, 1008–1015. doi: 10.1126/science.aah4967, PMID: 27885007 PMC7261592

[ref138] Patseadou M. Michala L. (2017). Usage of the levonorgestrel-releasing intrauterine system (LNG-IUS) in adolescence: what is the evidence so far? Arch. Gynecol. Obstet. 295, 529–541. doi: 10.1007/s00404-016-4261-0, PMID: 27928678

[ref139] Peachman R. R. (2018). Weighing the risks and benefits of hormonal contraception. JAMA 319, 1083–1084. doi: 10.1001/jama.2018.0448, PMID: 29490363

[ref140] Peña A. S. Doherty D. A. Atkinson H. C. Hickey M. Norman R. J. Hart R. (2018). The majority of irregular menstrual cycles in adolescence are ovulatory: results of a prospective study. Arch. Dis. Child. 103, 235–239. doi: 10.1136/archdischild-2017-312968, PMID: 28794095

[ref141] Pereira L. R. Moreira F. P. Reyes A. N. Bach S. L. Amaral P. L. Motta J. D. S.. (2019). Biological rhythm disruption associated with obesity in school children. Child. Obes. 15, 200–205. doi: 10.1089/chi.2018.0212, PMID: 30694701

[ref142] Piekarski D. J. Johnson C. M. Boivin J. R. Thomas A. W. Lin W. C. Delevich K.. (2017). Does puberty mark a transition in sensitive periods for plasticity in the associative neocortex? Brain Res. 1654, 123–144. doi: 10.1016/j.brainres.2016.08.042, PMID: 27590721 PMC5283387

[ref143] Piet R. Fraissenon A. Boehm U. Herbison A. E. (2015). Estrogen permits vasopressin signaling in preoptic kisspeptin neurons in the female mouse. J. Neurosci. 35, 6881–6892. doi: 10.1523/JNEUROSCI.4587-14.2015, PMID: 25926463 PMC6605180

[ref144] Pinkerton G. D. Carey H. M. (1976). Post-pill anovulation. Med. J. Aust. 1, 220–222. PMID: 1263979

[ref146] Polugrudov A. S. Panev A. S. Smirnov V. V. Paderin N. M. Borisenkov M. F. Popov S. V. (2016). Wrist temperature and cortisol awakening response in humans with social jetlag in the north. Chronobiol. Int. 33, 802–809. doi: 10.3109/07420528.2016.1168829, PMID: 27101215

[ref147] Prakapenka A. V. Hiroi R. Quihuis A. M. Carson C. Patel S. Berns-Leone C.. (2018). Contrasting effects of individual versus combined estrogen and progestogen regimens as working memory load increases in middle-aged ovariectomized rats: one plus one does not equal two. Neurobiol. Aging 64, 1–14. doi: 10.1016/j.neurobiolaging.2017.11.015, PMID: 29316527 PMC5820186

[ref148] Prendergast B. J. Beery A. K. Paul M. J. Zucker I. (2012). Enhancement and suppression of ultradian and circadian rhythms across the female hamster reproductive cycle. J. Biol. Rhythm. 27, 246–256. doi: 10.1177/0748730412441315, PMID: 22653893 PMC3965332

[ref149] Prendergast B. J. Zucker I. (2016). Ultradian rhythms in mammalian physiology and behavior. Curr. Opin. Neurobiol. 40, 150–154. doi: 10.1016/j.conb.2016.07.011, PMID: 27568859

[ref151] Pronina T. S. Orlova N. I. Rybakov V. P. (2015). Circadian rhythm of skin temperature of children during puberty. Fiziol. Cheloveka 41, 74–84. PMID: 26027336

[ref152] Refinetti R. (1994). Circadian modulation of ultradian oscillation in the body temperature of the golden hamster. J. Therm. Biol. 19, 269–275. doi: 10.1016/0306-4565(94)90050-7

[ref153] Righetti F. Tybur J. Van Lange P. Echelmeyer L. van Esveld S. Kroese J.. (2020). How reproductive hormonal changes affect relationship dynamics for women and men: a 15-day diary study. Biol. Psychol. 149:107784. doi: 10.1016/j.biopsycho.2019.107784, PMID: 31628974

[ref154] Russo K. A. La J. L. Stephens S. B. Z. Poling M. C. Padgaonkar N. A. Jennings K. J.. (2015). Circadian control of the female reproductive axis through gated responsiveness of the RFRP-3 system to VIP signaling. Endocrinology 156, 2608–2618. doi: 10.1210/en.2014-1762, PMID: 25872006 PMC4475714

[ref155] Sanchez-Alavez M. Alboni S. Conti B. (2011). Sex- and age-specific differences in core body temperature of C57Bl/6 mice. Age 33, 89–99. doi: 10.1007/s11357-010-9164-6, PMID: 20635153 PMC3063645

[ref156] Sanchez-Alavez M. Tabarean I. V. Osborn O. Mitsukawa K. Schaefer J. Dubins J.. (2010). Insulin causes hyperthermia by direct inhibition of warm-sensitive neurons. Diabetes 59, 43–50. doi: 10.2337/db09-1128, PMID: 19846801 PMC2797943

[ref157] Santoru F. Berretti R. Locci A. Porcu P. Concas A. (2014). Decreased allopregnanolone induced by hormonal contraceptives is associated with a reduction in social behavior and sexual motivation in female rats. Psychopharmacology 231, 3351–3364. doi: 10.1007/s00213-014-3539-9, PMID: 24728651

[ref158] Shannahoff-Khalsa D. S. Kennedy B. Yates F. E. Ziegler M. G. (1996). Ultradian rhythms of autonomic, cardiovascular, and neuroendocrine systems are related in humans. Am. J. Phys. 270, R873–R887. doi: 10.1152/ajpregu.1996.270.4.R873, PMID: 8967418

[ref159] Sharma A. Sethi G. Tambuwala M. M. Aljabali A. A. A. Chellappan D. K. Dua K.. (2021). Circadian rhythm disruption and alzheimer’s disease: the dynamics of a vicious cycle. Curr. Neuropharmacol. 19, 248–264. doi: 10.2174/1570159X18666200429013041, PMID: 32348224 PMC8033974

[ref160] Shaw N. D. Butler J. P. McKinney S. M. Nelson S. A. Ellenbogen J. M. Hall J. E. (2012). Insights into puberty: the relationship between sleep stages and pulsatile LH secretion. J. Clin. Endocrinol. Metab. 97, E2055–E2062. doi: 10.1210/jc.2012-2692, PMID: 22948756 PMC3485602

[ref161] Shechter A. Boivin D. B. (2010). Sleep, hormones, and circadian rhythms throughout the menstrual cycle in healthy women and women with premenstrual dysphoric disorder. Int. J. Endocrinol. 2010:e259345. doi: 10.1155/2010/259345, PMID: 20145718 PMC2817387

[ref162] Shechter A. Boudreau P. Varin F. Boivin D. B. (2011). Predominance of distal skin temperature changes at sleep onset across menstrual and circadian phases. J. Biol. Rhythm. 26, 260–270. doi: 10.1177/0748730411404677, PMID: 21628553

[ref163] Shilaih M. Goodale B. M. Falco L. Kübler F. De Clerck V. Leeners B. (2017). Modern fertility awareness methods: wrist wearables capture the changes of temperature associated with the menstrual cycle. Biosci. Rep. 38:BSR20171279. doi: 10.1042/BSR20171279, PMID: 29175999 PMC6265623

[ref164] Simone J. Bogue E. A. Bhatti D. L. Day L. E. Farr N. A. Grossman A. M.. (2015). Ethinyl estradiol and levonorgestrel alter cognition and anxiety in rats concurrent with a decrease in tyrosine hydroxylase expression in the locus coeruleus and brain-derived neurotrophic factor expression in the hippocampus. Psychoneuroendocrinology 62, 265–278. doi: 10.1016/j.psyneuen.2015.08.015, PMID: 26352480

[ref165] Sir-Petermann T. Piwonka V. Pérez F. Maliqueo M. Recabarren S. E. Wildt L. (1999). Are circulating leptin and luteinizing hormone synchronized in patients with polycystic ovary syndrome? Hum. Reprod. 14, 1435–1439. doi: 10.1093/humrep/14.6.1435, PMID: 10357954

[ref166] Sisk C. L. Foster D. L. (2004). The neural basis of puberty and adolescence. Nat. Neurosci. 7, 1040–1047. doi: 10.1038/nn1326, PMID: 15452575

[ref167] Skovlund C. W. Mørch L. S. Kessing L. V. Lange T. Lidegaard Ø. (2018). Association of hormonal contraception with suicide attempts and suicides. Am. J. Psychiatry 175, 336–342. doi: 10.1176/appi.ajp.2017.17060616, PMID: 29145752

[ref168] Skovlund C. W. Mørch L. S. Kessing L. V. Lidegaard Ø. (2016). Association of hormonal contraception with depression. JAMA Psychiatry 73, 1154–1162. doi: 10.1001/jamapsychiatry.2016.2387, PMID: 27680324

[ref169] Smarr B. L. Burnett D. C. Mesri S. M. Pister K. S. J. Kriegsfeld L. J. (2016a). A wearable sensor system with circadian rhythm stability estimation for prototyping biomedical studies. IEEE Trans. Affect. Comput. 7, 220–230. doi: 10.1109/TAFFC.2015.2511762

[ref170] Smarr B. L. Grant A. D. Zucker I. Prendergast B. J. Kriegsfeld L. J. (2017). Sex differences in variability across timescales in BALB/c mice. Biol. Sex Differ. 8:7. doi: 10.1186/s13293-016-0125-3, PMID: 28203366 PMC5301430

[ref171] Smarr B. L. Zucker I. Kriegsfeld L. J. (2016b). Detection of successful and unsuccessful pregnancies in mice within hours of pairing through frequency analysis of high temporal resolution core body temperature data. PLoS One 11:e0160127. doi: 10.1371/journal.pone.0160127, PMID: 27467519 PMC4965159

[ref172] Strittmatter E. Holtmann M. (2020). Gender identities in transition. Z. Kinder. Jugendpsychiatr. Psychother. 48, 93–102. doi: 10.1024/1422-4917/a000724, PMID: 32162593

[ref173] Strom J. O. Theodorsson E. Holm L. Theodorsson A. (2010). Different methods for administering 17β-estradiol to ovariectomized rats result in opposite effects on ischemic brain damage. BMC Neurosci. 11:39. doi: 10.1186/1471-2202-11-39, PMID: 20236508 PMC2848231

[ref174] Ström J. O. Theodorsson A. Ingberg E. Isaksson I.-M. Theodorsson E. (2012). Ovariectomy and 17β-estradiol replacement in rats and mice: a visual demonstration. J. Vis. Exp. 64:e4013. doi: 10.3791/4013, PMID: 22710371 PMC3471296

[ref175] Takahashi J. S. (2017). Transcriptional architecture of the mammalian circadian clock. Nat. Rev. Genet. 18, 164–179. doi: 10.1038/nrg.2016.150, PMID: 27990019 PMC5501165

[ref176] Tenreiro S. Dowse H. B. D’Souza S. Minors D. Chiswick M. Simms D.. (1991). The development of ultradian and circadian rhythms in premature babies maintained in constant conditions. Early Hum. Dev. 27, 33–52. doi: 10.1016/0378-3782(91)90026-Y, PMID: 1802663

[ref177] Touma C. Palme R. Sachser N. (2004). Analyzing corticosterone metabolites in fecal samples of mice: a noninvasive technique to monitor stress hormones. Horm. Behav. 45, 10–22. doi: 10.1016/j.yhbeh.2003.07.002, PMID: 14733887

[ref178] van der Vinne V. Pothecary C. A. Wilcox S. L. McKillop L. E. Benson L. A. Kolpakova J.. (2020). Continuous and non-invasive thermography of mouse skin accurately describes core body temperature patterns, but not absolute core temperature. Sci. Rep. 10:20680. doi: 10.1038/s41598-020-77786-5, PMID: 33244132 PMC7693264

[ref179] Veldhuis J. D. Christiansen E. Evans W. S. Kolp L. A. Rogol A. D. Johnson M. L. (1988). Physiological profiles of episodic progesterone release during the midluteal phase of the human menstrual cycle: analysis of circadian and ultradian rhythms, discrete pulse properties, and correlations with simultaneous luteinizing hormone release. J. Clin. Endocrinol. Metab. 66, 414–421. doi: 10.1210/jcem-66-2-414, PMID: 3339114

[ref180] Vidal J. D. (2017). The impact of age on the female reproductive system. Toxicol. Pathol. 45, 206–215. doi: 10.1177/0192623316673754, PMID: 27753638

[ref181] Violin Plots 101: Visualizing Distribution and Probability Density. (2016). Available at: https://mode.com/blog/violin-plot-examples/ (Accessed March 3, 2021).

[ref182] Walker J. J. Terry J. R. Tsaneva-Atanasova K. Armstrong S. P. McArdle C. A. Lightman S. L. (2010). Encoding and decoding mechanisms of pulsatile hormone secretion. J. Neuroendocrinol. 22, 1226–1238. doi: 10.1111/j.1365-2826.2010.02087.x, PMID: 21054582

[ref183] Wang F. Xie N. Wu Y. Zhang Q. Zhu Y. Dai M.. (2020). Association between circadian rhythm disruption and polycystic ovary syndrome. Fertil. Steril. 115, 771–781. doi: 10.1016/j.fertnstert.2020.08.1425, PMID: 33358334

[ref185] Webster W. W. Smarr B. (2020). Using circadian rhythm patterns of continuous core body temperature to improve fertility and pregnancy planning. J. Circadian Rhythms 18:5. doi: 10.5334/jcr.200, PMID: 33024445 PMC7518073

[ref186] Wegorzewska I. N. Walters K. Weiser M. J. Cruthirds D. F. Ewell E. Larco D. O.. (2008). Postovariectomy weight gain in female rats is reversed by estrogen receptor α agonist, propylpyrazoletriol. Am. J. Obstet. Gynecol. 199, 67.e1–67.e5. doi: 10.1016/j.ajog.2007.11.054, PMID: 18241818

[ref187] Westhoff C. L. Pike M. C. (2018). Hormonal contraception and breast cancer. Contraception 98, 171–173. doi: 10.1016/j.contraception.2018.05.002, PMID: 30193687 PMC6666389

[ref188] Williams H. Dacks P. A. Rance N. E. (2010). An improved method for recording tail skin temperature in the rat reveals changes during the estrous cycle and effects of ovarian steroids. Endocrinology 151, 5389–5394. doi: 10.1210/en.2010-0630, PMID: 20861232 PMC2954718

[ref189] Williams W. P. Jarjisian S. G. Mikkelsen J. D. Kriegsfeld L. J. (2011). Circadian control of kisspeptin and a gated GnRH response mediate the preovulatory luteinizing hormone surge. Endocrinology 152, 595–606. doi: 10.1210/en.2010-0943, PMID: 21190958 PMC3037169

[ref190] Woodruff J. A. Lacey E. A. Bentley G. (2010). Contrasting fecal corticosterone metabolite levels in captive and free-living colonial tuco-tucos (Ctenomys sociabilis). J. Exp. Zool. A Ecol. Genet. Physiol. 313A, 498–507. doi: 10.1002/jez.621, PMID: 20878749

[ref191] Wu F. C. Butler G. E. Kelnar C. J. Huhtaniemi I. Veldhuis J. D. (1996). Ontogeny of pulsatile gonadotropin releasing hormone secretion from midchildhood, through puberty, to adulthood in the human male: a study using deconvolution analysis and an ultrasensitive immunofluorometric assay. J. Clin. Endocrinol. Metab. 81, 1798–1805. doi: 10.1210/jcem.81.5.8626838, PMID: 8626838

[ref192] Zhang Z. Wang H.-J. Wang D.-R. Qu W.-M. Huang Z.-L. (2017). Red light at intensities above 10 lx alters sleep–wake behavior in mice. Light Sci. Appl. 6:e16231. doi: 10.1038/lsa.2016.231, PMID: 30167247 PMC6062196

[ref193] Zhou X. Shao Q. Han X. Weng L. Sang G. (1998). Pharmacokinetics of medroxyprogesterone acetate after single and multiple injection of cyclofem® in Chinese women. Contraception 57, 405–411. doi: 10.1016/S0010-7824(98)00048-1, PMID: 9693401

[ref194] Zimmet P. Alberti K. G. M. M. Stern N. Bilu C. El-Osta A. Einat H.. (2019). The circadian syndrome: is the metabolic syndrome and much more! J. Intern. Med. 286, 181–191. doi: 10.1111/joim.12924, PMID: 31081577 PMC6851668

