# Supplementary figures and images for: Adolescent Development of Biological Rhythms in Female Rats: Estradiol Dependence and Effects of Combined Contraceptives
Source: Front Physiol. 2021 Nov 5;12:752363. doi: 10.3389/fphys.2021.752363 (PMC9126190; doi:10.3389/fphys.2021.752363)

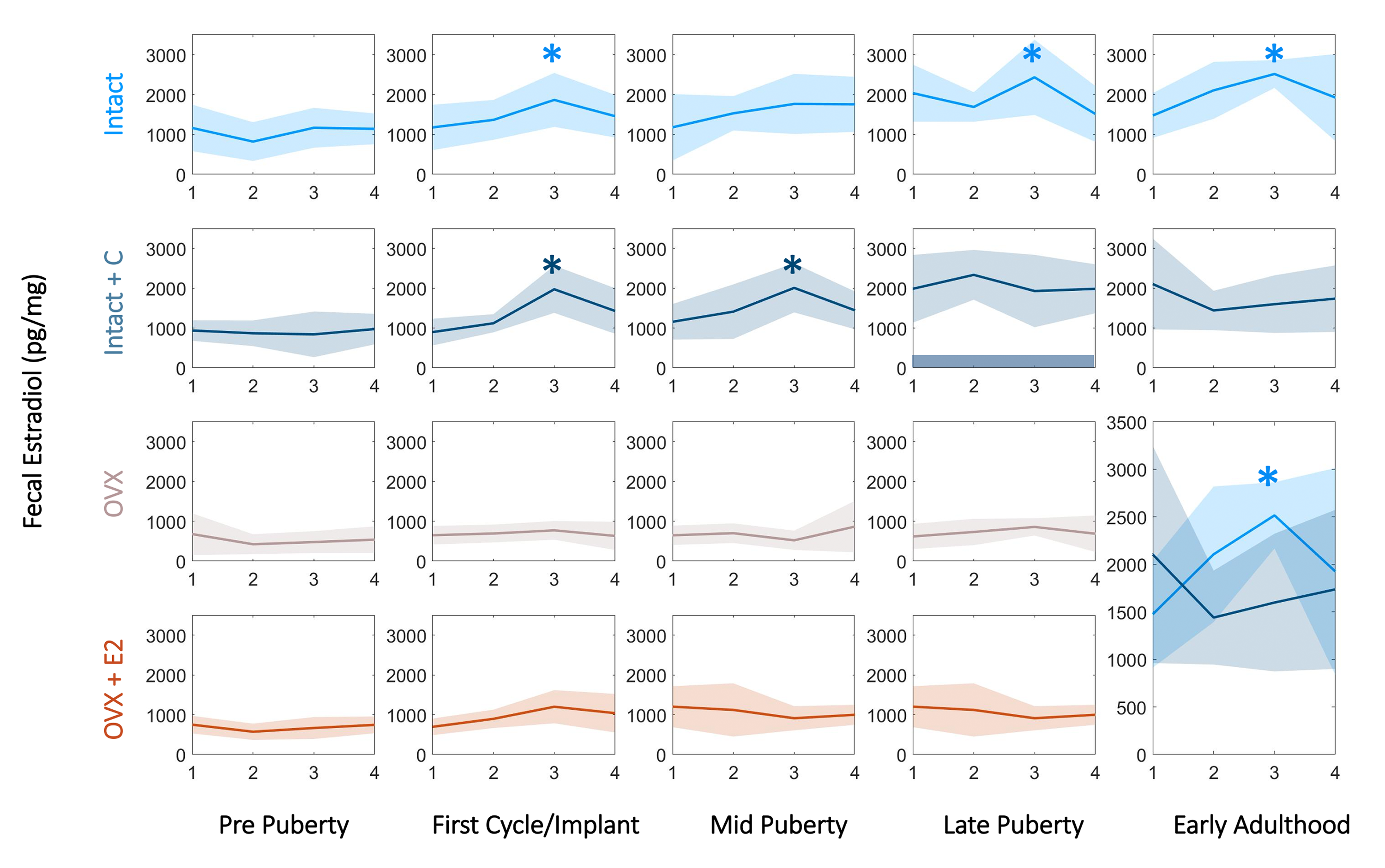

Supplement: Supplementary Figure 1 — Fecal estradiol (fE2) group averages from pre-puberty to adulthood post-contraception: contraceptives longitudinally alter the pattern but not level of fecal estradiol (fE2). Pre-puberty (p24–p30), early puberty (p30–p37), mid puberty (p43–p49), late puberty (p55–p61), and early adulthood (p69–p76) in Intact (A), Intact+C (B), OVX (C), and OVX+E2 (D). Early adulthood post-contraceptive administration, captured only in Intact and Intact+C animals, are overlaid in (E), illustrating persistent perturbation of estrous-cycle fE2 patterning. Dark bar indicates samples were collected during contraceptive administration in Intact+C animals. * indicates significant elevation of day 3 compared to day 1 (p<0.05). [file Figure5.tif]

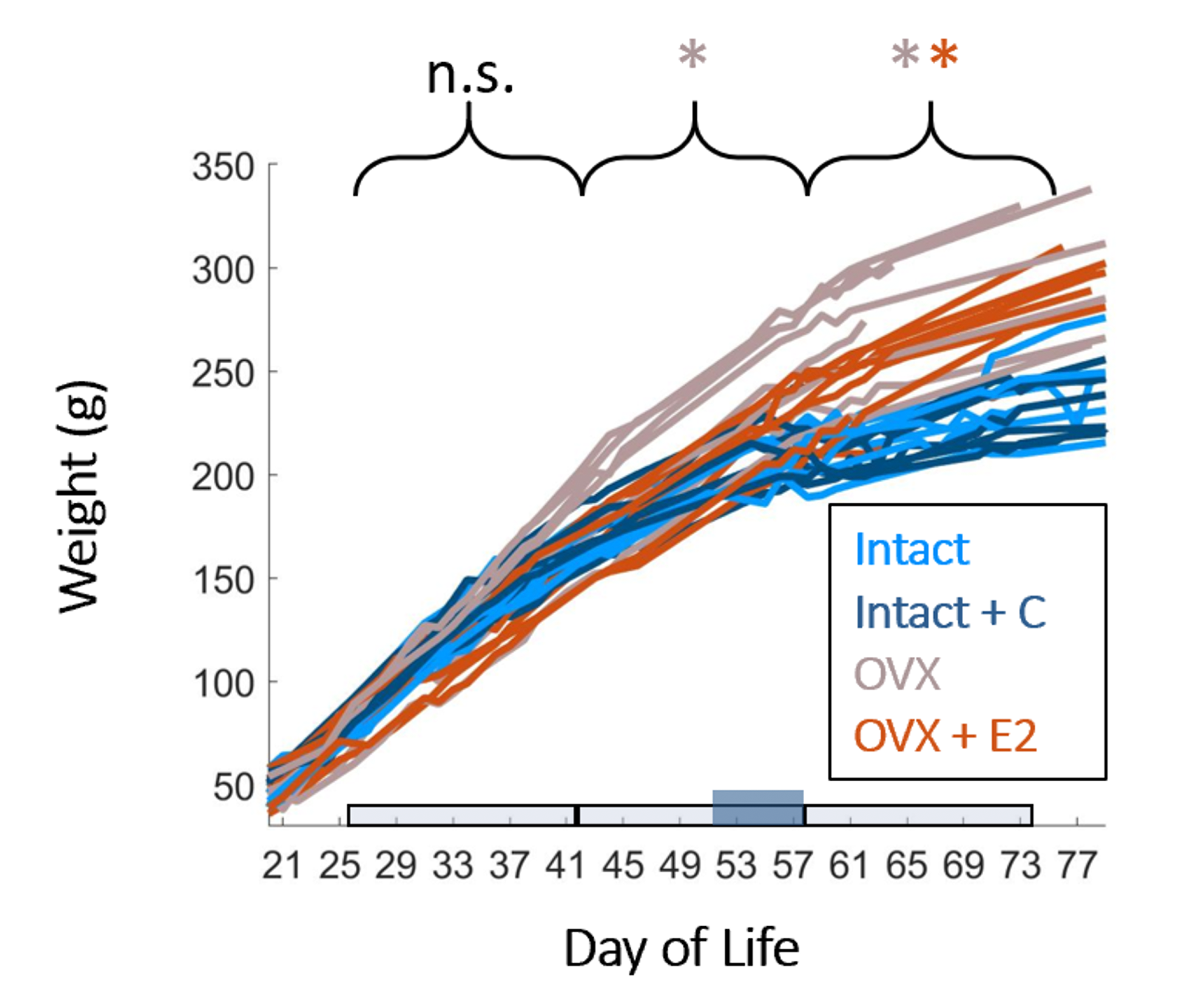

Supplement: Supplementary Figure 2 — Ovariectomized (OVX) and OVX+E2 are heavier than Intact animals in mid puberty and early adulthood. Prepubertal OVX did not significantly impact weight gain before mid-puberty. OVX animals (gray) are significantly heavier than all other groups at pre to mid-puberty. OVX animals remain significantly heavier into adulthood. In early adulthood, OVX+E2 animals (orange) are not different from OVX animals and are significantly heavier than either Intact (light blue) or Intact+C animals (dark blue). Colors of * indicate groups that are significantly heavier (p<0.05). [file Figure6.tif]

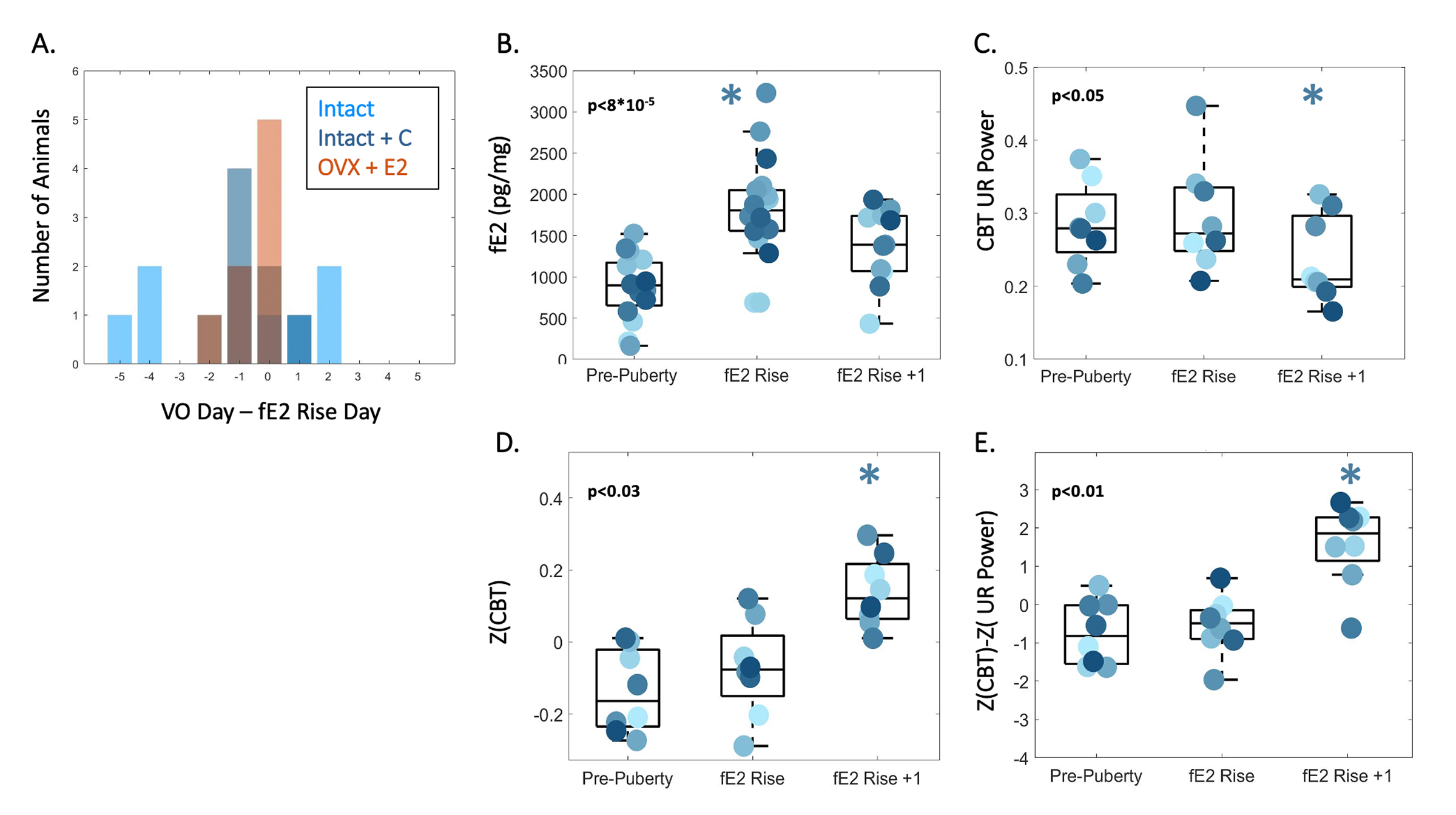

Supplement: Supplementary Figure 3 — Day of first estradiol rise occurs near vaginal opening and coincides with markers of estrus: subsequent drop in ultradian rhythm (UR) power and rise in core body temperature (CBT). Estradiol has a unique day in proximity to, but distinct from, vaginal opening, during which fE2 rises >2 SDs within an individual (A,B). Alignment to this day as a proxy for puberty onset provides a convenient point from which to average across animals to permit easy visualization of the estrous cycle. Daily mean UR power decreases significantly following this day (C), and daily mean CBT rises significantly following this day (D). The difference between these two metrics exaggerates the difference and the differences across subsequent cycles (E). After this point, all three metrics commence the 4-day estrous pattern. * indicates significant difference from all other time periods. [file Figure7.tif]

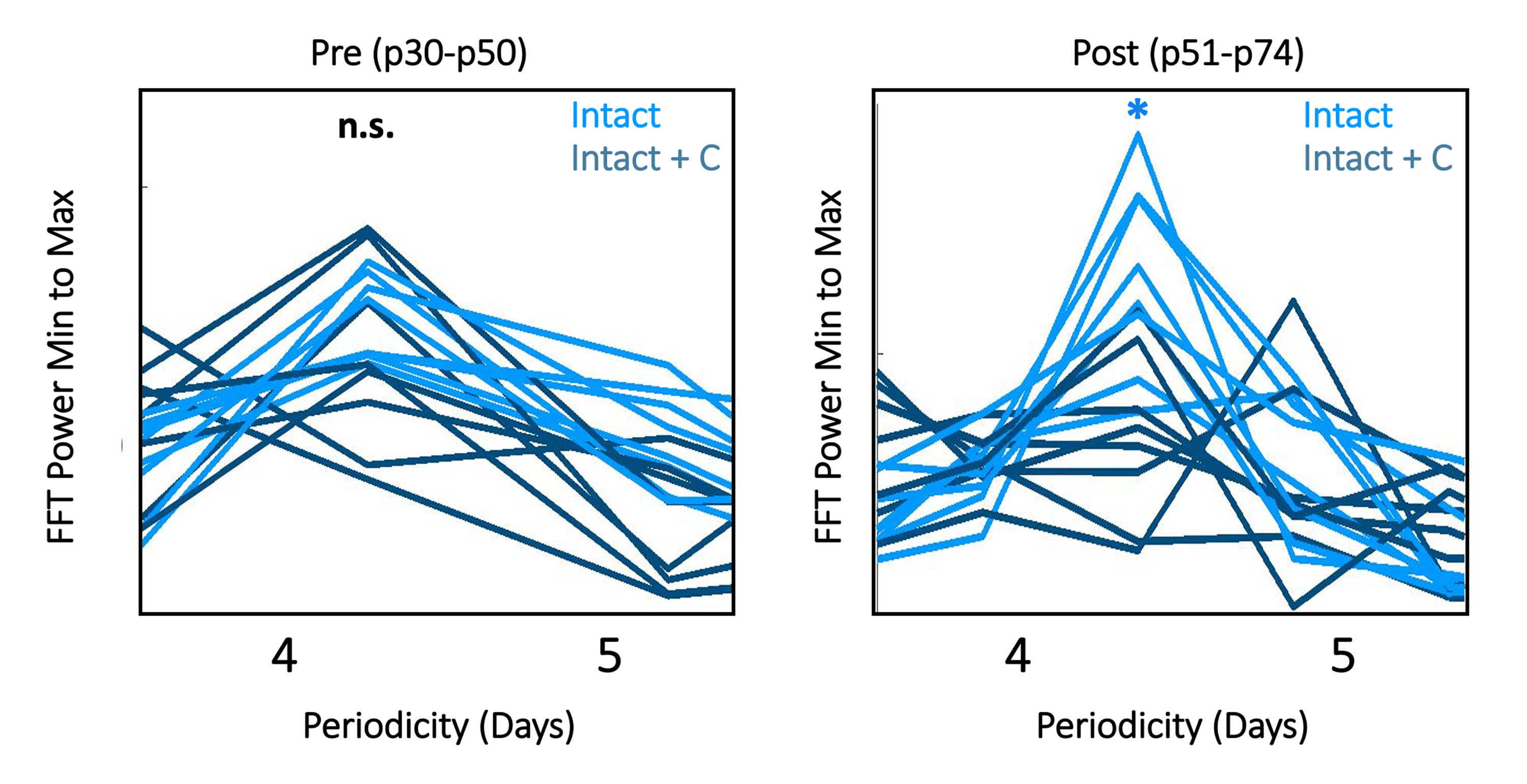

Supplement: Supplementary Figure 4 — Fast Fourier Transform (FFT) power of Intact temperature across life peaks unique at 4days and is disrupted during and after contraceptive administration. Fast Fourier Transform of each individual’s CBT log in Intact (light blue) and Intact+BC (dark blue) prior to contraceptive administration (left), and during and after contraceptive administration (right). * indicates significant difference for AUC between the groups in 4–5 day periodicity. [file Figure8.tif]

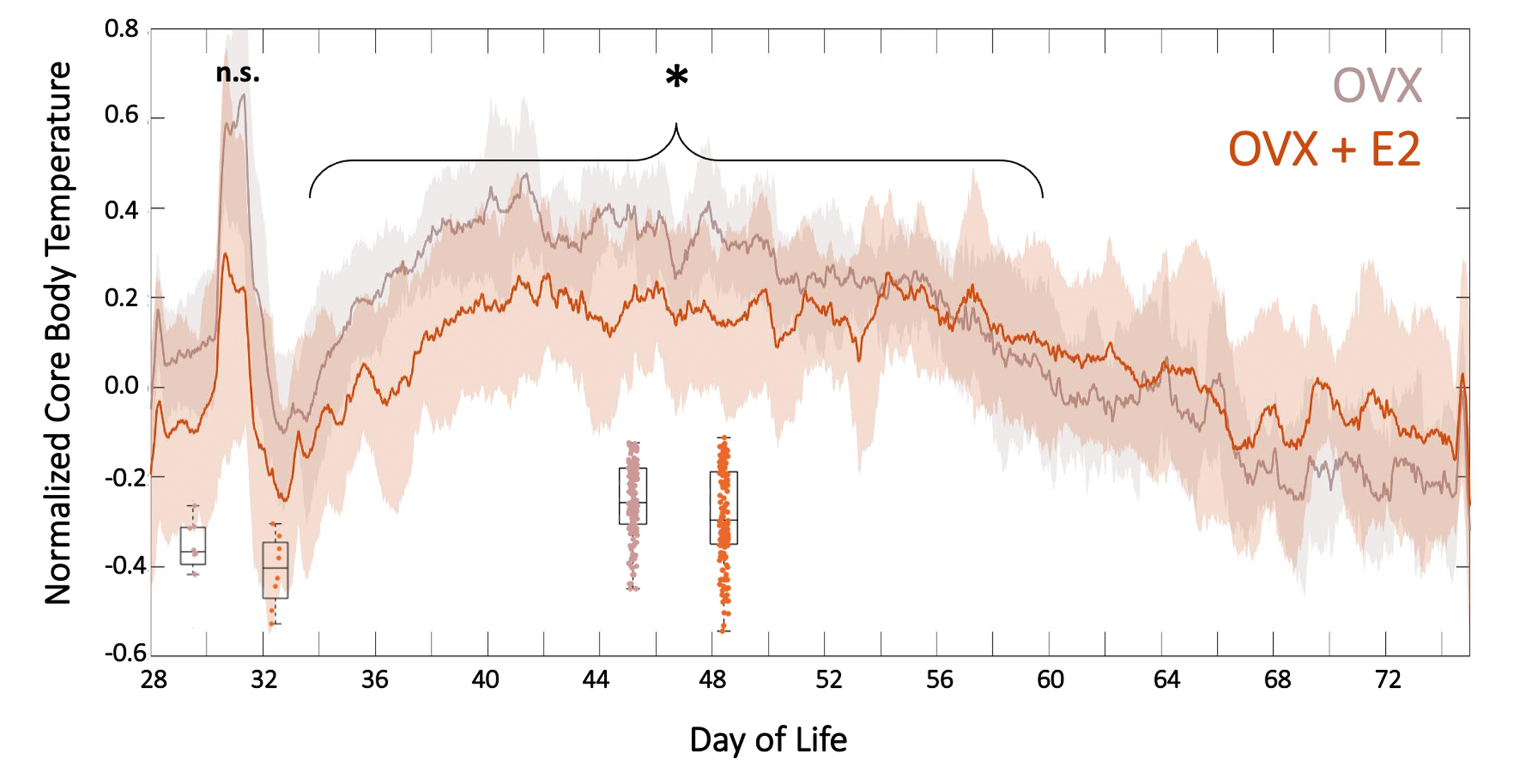

Supplement: Supplementary Figure 5 — Estradiol administration in ovariectomized animals is associated with lower temperatures across adolescence. Core CBT linear group means (±SD) in OVX (gray) and OVX+E2 animals from p28 to p72. Box plots indicate values used in Kruskal-Wallis (KW) comparisons between groups during silastic implant recovery (left) and across adolescence (right) with independent y-axes. * indicates significant difference between groups during the marked time period (p<0.03). [file Figure9.tif]

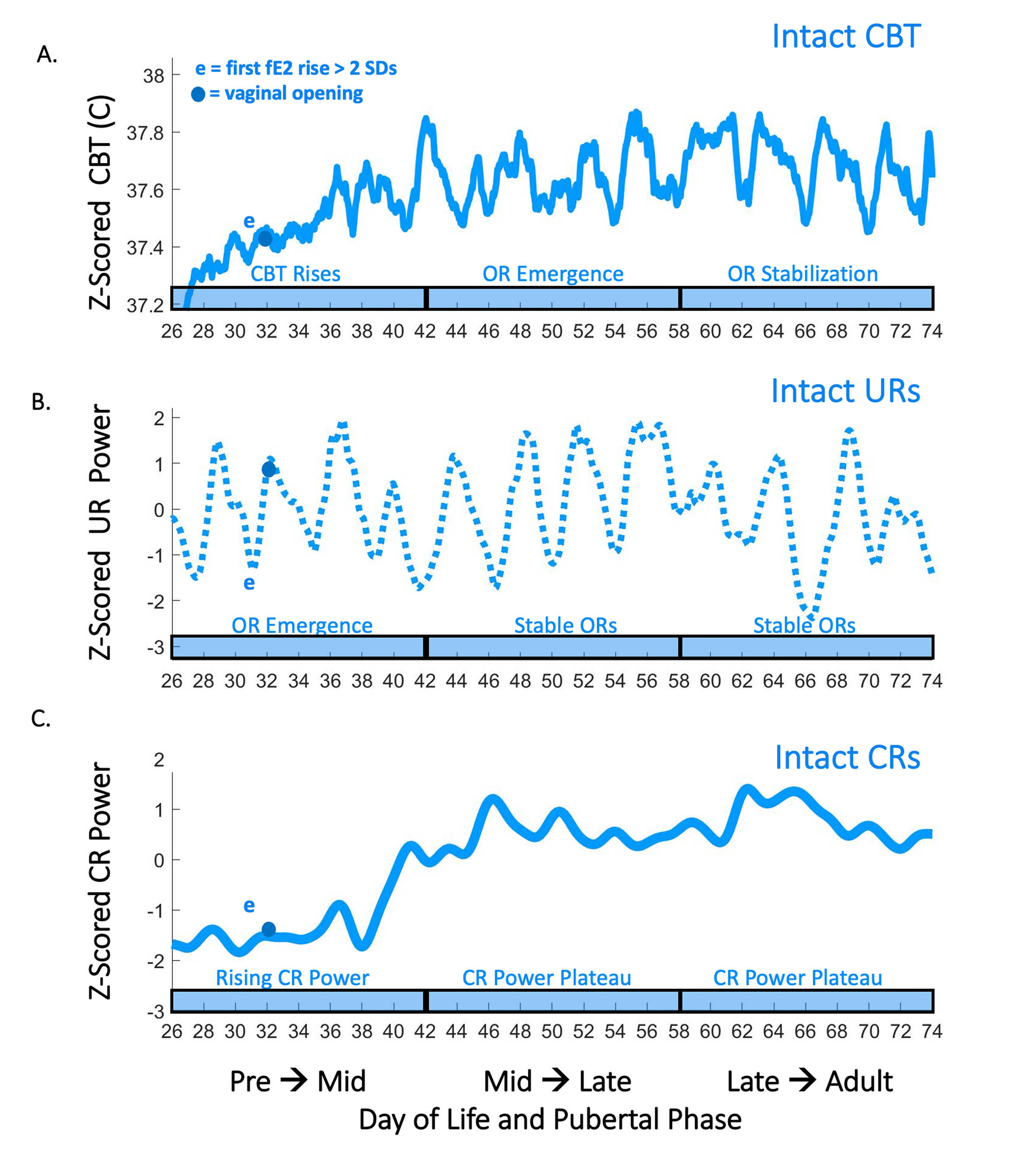

Supplement: Supplementary Figure 6 — Summary of CBT feature use for pubertal staging in an Intact female. Features of CBT and CBT rhythmicity can be used to complement and extend hormonal and external markers of adolescence. Here, we illustrate derived features on an example Intact female from p26 to p74, with fE2 rise and vaginal opening occurring on p31 and p32, respectively. Dots indicate vaginal opening and “e” indicates first rise of fE2 >2 SDs. Rising CBT, emergence of ovulatory rhythms (ORs) in UR power, and rising circadian rhythm (CR) power can be used to characterize pubertal onset. Mid to late adolescence is characterized by a plateau of CR power, as well as the emergence of ORs in raw CBT their persistence in UR power. Post-natal day 60 did not correspond with any overt transitions in CBT or CBT metrics. [file Figure10.tif]

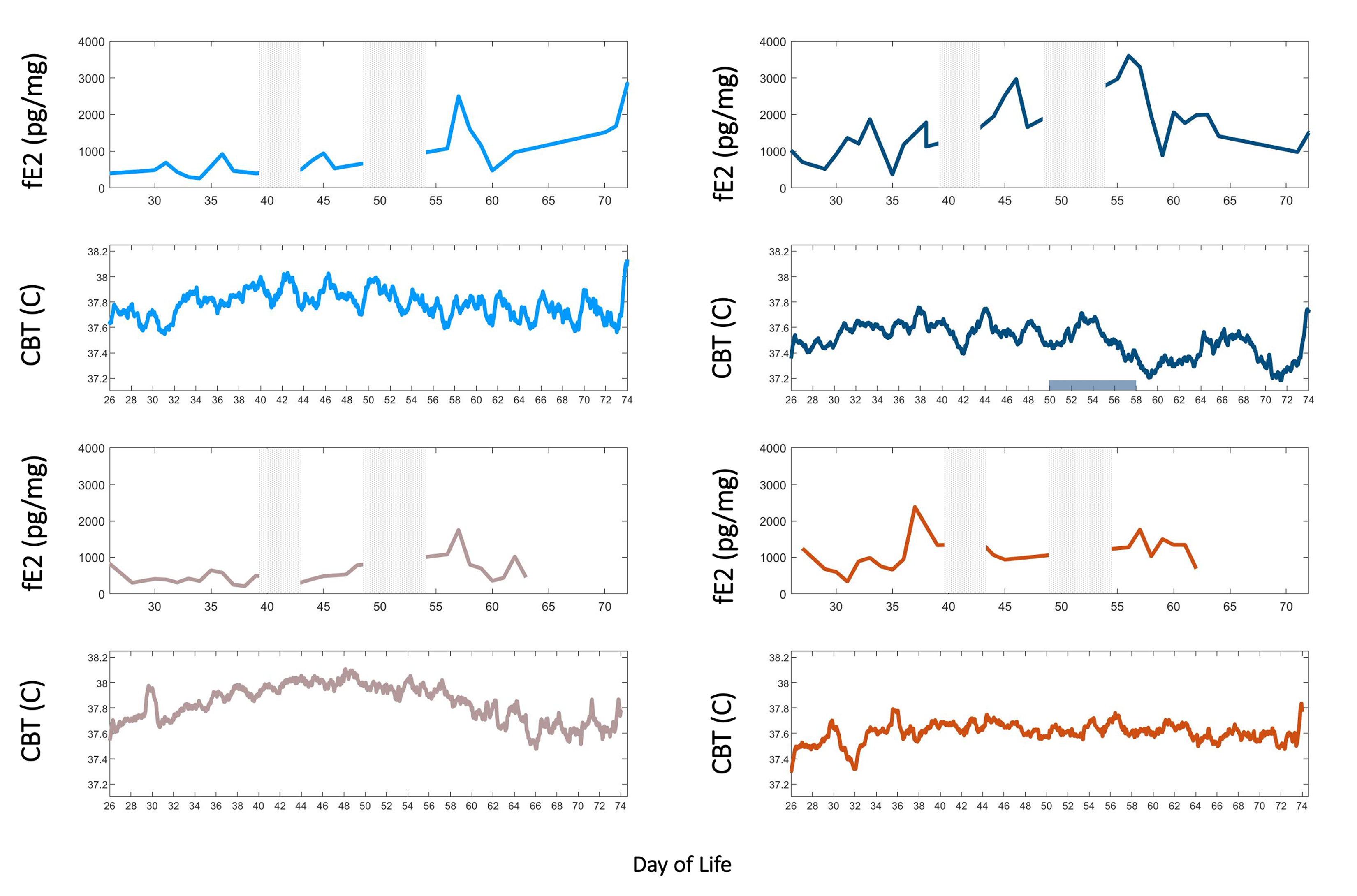

Supplement: Supplementary Figure 7 — Examples of individual fE2 and CBT. Representative individual plots of fE2 (top) and CBT (bottom) from Intact (A), Intact+C (B), OVX (C), and OVX+E2 animals. Gray bars indicate windows of time during which fE2 was not collected. Horizontal bar in B indicates days during which hormonal contraception was administered. [file Figure11.tif]
